# Vanzacaftor–tezacaftor–deutivacaftor versus elexacaftor–tezacaftor–ivacaftor in individuals with cystic fibrosis aged 12 years and older (SKYLINE Trials VX20–121-102 and VX20–121-103): results from two randomised, active-controlled, phase 3 trials

**DOI:** 10.1016/S2213-2600(24)00411-9

**Published:** 2025-01-02

**Authors:** Claire Keating, Lael M Yonker, François Vermeulen, Dario Prais, Rachel W Linnemann, Aaron Trimble, Tom Kotsimbos, Joel Mermis, Andrew T Braun, Mark O’Carroll, Sivagurunathan Sutharsan, Bonnie Ramsey, Marcus A Mall, Jennifer L Taylor-Cousar, Edward F McKone, Elizabeth Tullis, Tim Floreth, Peter Michelson, Patrick R Sosnay, Nitin Nair, Rachel Zahigian, Hannah Martin, Neil Ahluwalia, Anna Lam, Alexander Horsley

**Affiliations:** Columbia University Irving Medical Center, New York, NY, USA (C Keating MD); Massachusetts General Hospital, Boston, MA, USA (L M Yonker MD); Cystic Fibrosis Reference Centre, Department of Pediatrics, Katholieke Universiteit Leuven, Leuven, Belgium (F Vermeulen MD); Pediatric Pulmonology Institute, Schneider Children’s Medical Center and Faculty of Medical and Health Sciences, Tel Aviv University, Tel Aviv, Israel (D Prais MD); Emory University, Children’s Healthcare of Atlanta, Atlanta, GA, USA (R W Linnemann MD); Oregon Health and Science University, Portland, OR, USA (A Trimble MD); Alfred Hospital, Monash University Melbourne, Melbourne, VIC, Australia (T Kotsimbos MD); University of Kansas Medical Center, Kansas City, KS, USA (J Mermis MD); Department of Medicine, Division of Pulmonary and Critical Care Medicine, University of Wisconsin School of Medicine and Public Health, Madison, WI, USA (A T Braun MD); Auckland City Hospital, Health New Zealand, Auckland, New Zealand (M O’Carroll MBChB); Department of Pulmonary Medicine, Division of Cystic Fibrosis, University Medicine Essen-Ruhrlandklinik, University of Duisburg-Essen, Essen, Germany (S Sutharsan MD); Seattle Children’s Hospital, University of Washington, Seattle, WA, USA (Prof B Ramsey MD); Department of Pediatric Respiratory Medicine, Immunology and Critical Care Medicine, Charité - Universitätsmedizin Berlin, Berlin, Germany (Prof M A Mall MD); German Center for Lung Research (DZL), associated partner site Berlin, Berlin, Germany (Prof M A Mall); German Center for Child and Adolescent Health (DZKJ), partner site, Berlin, Germany (Prof M A Mall); National Jewish Health, Denver, CO, USA (Prof J L Taylor-Cousar MD); St Vincent’s University Hospital, University College Dublin, Dublin, Ireland (E F McKone MD); St Michael’s Hospital, University of Toronto, Toronto, ON, Canada (Prof E Tullis MD); Vertex Pharmaceuticals, Boston, MA, USA (T Floreth MD, P Michelson MD, P R Sosnay MD, N Nair PhD, R Zahigian PhD, H Martin MD, N Ahluwalia MD, A Lam MD); Division of Infection, Immunity and Respiratory Medicine, University of Manchester, Manchester, UK (Prof A Horsley PhD)

## Abstract

**Background:**

The goal of cystic fibrosis transmembrane conductance regulator (CFTR) modulators is to reach normal CFTR function in people with cystic fibrosis. Vanzacaftor–tezacaftor–deutivacaftor restored CFTR function in vitro and in phase 2 trials in participants aged 18 years and older resulting in improvements in CFTR function, as measured by sweat chloride concentrations and lung function as measured by spirometry. We aimed to evaluate the efficacy and safety of vanzacaftor–tezacaftor–deutivacaftor compared with standard of care elexacaftor–tezacaftor–ivacaftor in individuals with cystic fibrosis aged 12 years and older.

**Methods:**

In two randomised, active-controlled, double-blind, phase 3 trials, individuals aged 12 years and older with stable cystic fibrosis with *F508del*-minimal function (SKYLINE Trial VX20–121-102) or with *F508del-F508del, F508del*-residual function, *F508del*-gating, or elexacaftor–tezacaftor–ivacaftor-responsive-non-*F508del* genotypes (SKYLINE Trial VX20–121-103) were enrolled at 126 and 159 international sites, respectively. Eligible individuals were entered into a 4-week run-in period, during which they received elexacaftor (200 mg once daily), tezacaftor (100 mg once daily), and ivacaftor (150 mg once every 12 h) as two fixed-dose combination tablets in the morning and one ivacaftor tablet in the evening. They were then randomly assigned (1:1) to either elexacaftor (200 mg once daily), tezacaftor (100 mg once daily), and ivacaftor (150 mg once every 12 h) as two fixed-dose combination tablets in the morning and one ivacaftor tablet in the evening, or vanzacaftor (20 mg once daily), tezacaftor (100 mg once daily), and deutivacaftor (250 mg once daily) as two fixed-dose combination tablets in the morning, for the 52-week treatment period. All participants received matching placebo tablets to maintain the treatment blinding. Randomisation was done using an interactive web-response system and stratified by age, FEV_1_ % predicted, sweat chloride concentration, and previous CFTR modulator use, and also by genotype for Trial VX20–121-103. The primary endpoint for both trials was absolute change in FEV_1_ % predicted from baseline (most recent value before treatment on day 1) through week 24 (with non-inferiority of vanzacaftor–tezacaftor–deutivacaftor shown if the lower bound of the 95% CI for the primary endpoint was –3·0 or higher). Efficacy was assessed in all participants with the intended *CFTR* genotype who were randomly assigned to treatment and received at least one dose of study treatment during the treatment period. Safety was assessed in all participants who received at least one dose of study drug during the treatment period. These trials are registered with ClinicalTrials.gov, NCT05033080 (Trial VX20–121-102) and NCT05076149 (Trial VX20–121-103), and are now complete.

**Findings:**

In Trial VX20–121-102 between Sept 14, 2021, and Oct 18, 2022, 488 individuals were screened, of whom 435 entered the 4-week run-in period, and subsequently 398 were randomly assigned and received at least one dose of elexacaftor–tezacaftor–ivacaftor (n=202) or vanzacaftor–tezacaftor–deutivacaftor (n=196). Median age was 31·0 years (IQR 22·6–38·5), 163 (41%) of 398 participants were female, 235 (59%) were male, and 388 (97%) were White. In Trial VX20–121-103, between Oct 27, 2021, and Oct 26, 2022, 699 individuals were screened, of whom 597 entered the 4-week run-in period, and subsequently 573 participants were randomly assigned and received at least one dose of elexacaftor–tezacaftor–ivacaftor (n=289) or vanzacaftor–tezacaftor–deutivacaftor (n=284). Median age was 33·1 years (IQR 24·5–42·2), 280 (49%) of 573 participants were female, 293 (51%) were male, and 532 (93%) were White. The absolute change in least squares mean FEV_1_ % predicted from baseline through week 24 for Trial VX20–121-102 was 0·5 (SE 0·3) percentage points in the vanzacaftor–tezacaftor–deutivacaftor group versus 0·3 (0·3) percentage points in the elexacaftor–tezacaftor–ivacaftor group (least squares mean treatment difference of 0·2 percentage points [95% CI –0·7 to 1·1]; p<0·0001), and for Trial VX20–121-103, was 0·2 (SE 0·3) percentage points in the vanzacaftor–tezacaftor–deutivacaftor group versus 0·0 (0·2) percentage points in the elexacaftor–tezacaftor–ivacaftor group (least squares mean treatment difference 0·2 percentage points [95% CI –0·5 to 0·9]; p<0·0001). Most adverse events were mild or moderate, with the most common being infective pulmonary exacerbation (133 [28%] of 480 participants in the pooled vanzacaftor–tezacaftor–deutivacaftor group *vs* 158 [32%] of 491 in the pooled elexacaftor–tezacaftor–ivacaftor group), cough (108 [23%] *vs* 101 [21%]), COVID-19 (107 [22%] *vs* 127 [26%]), and nasopharyngitis (102 [21%] *vs* 95 [19%]).

**Interpretation:**

Vanzacaftor–tezacaftor–deutivacaftor is non-inferior to elexacaftor–tezacaftor–ivacaftor in terms of FEV_1_ % predicted, and is safe and well tolerated. Once daily dosing with vanzacaftor–tezacaftor–deutivacaftor reduces treatment burden, potentially improving adherence, compared with the twice daily regimen of the current standard of care. The restoration of CFTR function and the potential variants treated are also considerations that should be compared with currently available CFTR modulators.

**Funding:**

Vertex Pharmaceuticals.

## Introduction

Cystic fibrosis is a multi-organ disease caused by dysfunction of the cystic fibrosis transmembrane conductance regulator (CFTR) protein. CFTR modulators target the underlying cause of cystic fibrosis and improve CFTR function with the goal of reaching normal levels of CFTR function in people with cystic fibrosis, potentially preventing disease progression.^1^Elexacaftor–tezacaftor–ivacaftor, the standard of care for eligible people with cystic fibrosis, provides clinically significant improvement in CFTR function (measured by concentrations of sweat chloride) leading to transformative benefits in people with cystic fibrosis with responsive *CFTR* variants (as identified through clinical and in-vitro data), including improvements in lung function, longer life expectancy, and reduction in lung transplant rates.^2–5^

In cystic fibrosis trials, clinical response is primarily assessed through lung function (FEV_1_ % predicted). Consistent with this standard, FEV_1_ % predicted continues to be the primary endpoint used by regulators to assess CTFR modulator therapies and remains of clinical interest for health-care providers and people with cystic fibrosis. Additionally, it is well established that CFTR dysfunction leads to higher concentrations of chloride in secreted sweat. Given that the sweat gland does not undergo damage from cystic fibrosis as is seen for other organ tissues, measurement of the concentration of chloride in sweat provides a stable measurement of underlying CFTR function.^6,7^ As such, sweat chloride is a sensitive and direct measure of CFTR function that is used in the diagnosis of cystic fibrosis and as a key secondary endpoint in clinical trials of CFTR modulators.^8,9^ A natural history study showed better survival and nutritional status, and slower rate of decline in lung function in people with cystic fibrosis with better CFTR function (sweat chloride concentration <60 mmol/L; below the diagnostic threshold of cystic fibrosis) than in those with worse CFTR function (sweat chloride concentration ≥60 mmol/L).^9,10^ Sweat chloride concentrations less than 30 mmol/L indicate normal CFTR function and are seen in carriers of *CFTR* variants who do not have signs or symptoms of cystic fibrosis disease and have a lifespan similar to the general population.^11,12^ Population-level improvements in sweat chloride concentrations are correlated with improvements in FEV_1_ % predicted across multiple interventional and observational studies.^4,13^ A retrospective analysis of data across multiple CFTR modulator trials found that greater decreases in sweat chloride concentrations after treatment with a CTFR modulator were associated with improved clinical outcomes (unpublished).^14^ These outcomes included improved FEV_1_ % predicted, Cystic Fibrosis Questionnaire-Revised (CFQ-R; a validated cystic fibrosis-specific quality-of-life measure) respiratory domain score, BMI, annual rate of change in FEV_1_ % predicted, and a lower annual rate of pulmonary exacerbations in participants who had sweat chloride concentrations below 60 mmol/L or below 30 mmol/L than those with concentrations of 60 mmol/L or higher.

Although elexacaftor–tezacaftor–ivacaftor provided clinically significant improvements in FEV_1_ % predicted and sweat chloride in clinical trials, a substantial subset of participants treated with elexacaftor–tezacaftor–ivacaftor did not reach normal sweat chloride concentrations and might therefore derive additional clinical benefit from more efficacious modulator therapies.^2,15^ Furthermore, an important requirement for eligibility for CFTR modulator therapy includes the presence of a *CFTR* genotype that has been shown to be responsive on the basis of clinical or in-vitro data. Some people with cystic fibrosis have genotypes that are not currently approved for CFTR modulator treatment, including with elexacaftor–tezacaftor–ivacaftor. Because assessment of every rare *CFTR* variant is not possible in clinical trials, in-vitro cell-based assays have been used to positively predict clinical response and support inclusion of additional variants in approved indications for CFTR modulators. Finally, there are people with cystic fibrosis who have discontinued elexacaftor–tezacaftor–ivacaftor due to a variety of reasons and have an unmet need for another CFTR modulator treatment option. Vanzacaftor–tezacaftor–deutivacaftor is a novel, once-daily CFTR modulator regimen that improved CFTR function compared with tezacaftor–ivacaftor in vitro and in vivo in phase 2 trials,^16^ supporting evaluation of efficacy and safety in phase 3 trials. The comprehensive clinical development programme of vanzacaftor–tezacaftor–deutivacaftor includes two randomised phase 3 trials in adolescents and adults (ie, aged ≥12 years) and one single-arm phase 3 trial in children (results from children aged 6–11 years reported simultaneously).^17^ Here we present data from the two randomised, active-controlled, phase 3 trials that evaluated the efficacy and safety of vanzacaftor–tezacaftor–deutivacaftor compared with elexacaftor–tezacaftor–ivacaftor in participants with cystic fibrosis aged 12 years and older.

## Methods

### Study design and participants

SKYLINE Trial VX20–121–102 and Trial VX20–121-103 were both multicentre, randomised, active-controlled, phase 3 trials, with Trial VX20–121-102 being conducted at 126 sites in Australia, Czech Republic, Germany, Hungary, Ireland, Israel, New Zealand, Portugal, Spain, Sweden, the UK, and the USA and Trial VX20–121-103 being conducted at 159 sites in Australia, Austria, Belgium, Canada, Denmark, France, Germany, Greece, Hungary, Ireland, Israel, Italy, Netherlands, New Zealand, Norway, Poland, Sweden, Switzerland, the UK, and the USA (appendix pp 32–38).

Trial VX20–121-102 enrolled individuals with cystic fibrosis with *F508del*-minimal function genotypes and Trial VX20–121-103 enrolled individuals with cystic fibrosis with *F508del-F508del*, *F508del*-residual func tion, *F508del*-gating, or elexacaftor–tezacaftor–ivacaftor-responsive-non-*F508del* genotypes. Individuals who had previously been unable to tolerate elexacaftor–tezacaftor–ivacaftor were excluded from the trials. Eligible partic ipants were aged 12 years and older with a confirmed diagnosis of cystic fibrosis with eligible genotypes and stable cystic fibrosis, as judged by the investigators. FEV_1_ % predicted at screening had to be 40–80% for those who had not previously received elexacaftor–tezacaftor–ivacaftor, or 40–90% for those who were receiving elexacaftor–tezacaftor–ivacaftor. All participants and their parents or legal guardian or caregivers, as applicable, agreed for the participant to continue their usual cystic fibrosis symptomatic medication regimens throughout the trial period (details of individual medication regimens were not collected after baseline). Complete eligibility criteria for each trial are in the appendix (pp 11–15). Data on sex were self-reported by the participant (or their parents or legal guardian or caregiver). Data on ethnicity and race were collected on the basis of the participant’s (or their parents or legal guardian or caregiver’s, as applicable) self-identification when allowable by local regulations.

An independent review board or ethics committee for each site approved the trial protocol and informed consent forms (central independent review board: Advarra Incorporated; approval numbers were PRO00054586 for Trial VX20–121-102 and Pro00054721 for Trial VX20–121-103; additional information is provided in the appendix pp 39–40). All enrolled participants, or their legal guardians, provided written informed consent (and signed assent). These trials are registered with ClinicalTrials.gov, NCT05033080 (Trial VX20–121-102) and NCT05076149 (Trial VX20–121-103), and are complete; trial protocols are available online

### Randomisation and masking

To establish a reliable on-treatment baseline, all par ticipants received elexacaftor–tezacaftor–ivacaftor during a 4-week run-in period. Participants were then randomly assigned (1:1) to vanzacaftor–tezacaftor–deutivacaftor or elexacaftor–tezacaftor–ivacaftor for the 52-week treatment period, after which participants had the opportunity to participate in an open-label extension study in which all participants received open-label vanzacaftor–tezacaftor–deutivacaftor, regardless of randomly assigned treatment in Trials VX20–121-102 and VX20–121-103.

For both trials, the same third-party vendor (Cytel, Cambridge, MA, USA) generated random code lists and participants were randomly assigned by means of an interactive web-response system. Randomisation was stratified on the basis of age (at screening: <18 years *vs* ≥18 years), FEV_1_ % predicted (on day –14: <70 percentage points *vs* ≥70 percentage points), sweat chloride concentration (on day –14: <30 mmol/L *vs* ≥30 mmol/L), previous CFTR modulator use (yes *vs* no), and, additionally for Trial VX20–121-103, by genotype group (*F508del-F508del vs F508del*-residual function *vs F508del*-gating *vs* elexacaftor–tezacaftor–ivacaftor-responsive-non-*F508del* genotypes). All participants, site personnel, and the sponsor’s study team were masked to treatment group assignment, and matching placebo tablets in size and appearance were given to maintain masking.

### Procedures

In the 4-week run-in period, all participants received elexacaftor 200 mg once daily, tezacaftor 100 mg once daily, and ivacaftor 150 mg once every 12 h as two fixed-dose combination tablets in the morning and one ivacaftor tablet in the evening (manufactured by Vertex Pharmaceuticals, Boston, MA, USA, and Patheon Pharmaceuticals, Cincinnati, OH, USA).

Starting on day 1 of the trial treatment period, participants received either elexacaftor 200 mg once daily, tezacaftor 100 mg once daily, and ivacaftor 150 mg once every 12 h as two fixed-dose combination tablets in the morning and one ivacaftor tablet in the evening (manufactured by Vertex Pharmaceuticals, Boston, MA, USA, and Patheon Pharmaceuticals, Cincinnati, OH, USA); or vanzacaftor 20 mg once daily, tezacaftor 100 mg once daily, and deutivacaftor 250 mg once daily as two fixed-dose combination tablets in the morning (manufactured by Patheon Pharmaceuticals, Cincinnati, OH, USA). All participants received matching placebo tablets to maintain treatment blinding (manufactured by Patheon Pharmaceuticals, Cincinnati, OH, USA, and Patheon Pharmaceuticals, Whitby, ON, Canada). Participants were treated for 52 weeks. Additional details for the trial designs, including stopping rules, discontinuation and interruption criteria, and the schedule and procedures for endpoint and safety measurements are in the appendix (pp 15–19).

The Fischer rat thyroid (FRT) system is a clinically validated in-vitro assay that measures CFTR-mediated chloride transport to assess responsiveness of *CFTR* variants to CFTR modulators. The FRT system is a stable expression system integrating mutated *CFTR* cDNA as a single copy into the same genomic location using the Invitrogen Flp-In system (Thermo Fisher Scientific, Waltham, MA, USA). Ussing chamber studies of cells stably expressing a *CFTR* variant were used to measure responsiveness of 128 variants to vanzacaftor–tezacaftor–deutivacaftor. Additional details and results are in the appendix (pp 10, 27).

### Outcomes

The primary endpoint for both trials was absolute change in FEV_1_ % predicted from elexacaftor–tezacaftor–ivacaftor baseline (defined as the most recent non-missing measurement before the first dose of study drug on day 1) through week 24 (estimated by averaging week 16 and week 24). Key secondary endpoints for both trials were tested for superiority in the following hierarchical testing order: absolute change from baseline through week 24 in sweat chloride concentration, proportion of participants with sweat chloride concentration below 60 mmol/L (pooled from both trials), and proportion of participants with sweat chloride concentration below 30 mmol/L (pooled from both trials) through week 24. For endpoints evaluating sweat chloride concentration, baseline was defined as the average of the two most recent pre-dose non-missing values on or after day –14, including unscheduled visits.

Other secondary endpoints (not included in the multiplicity-controlled testing hierarchy) for both trials were absolute change from baseline through week 24 in CFQ-R respiratory domain score, number of protocol-defined pulmonary exacerbations through week 52 (defined in the appendix [pp 22–23]), proportion of participants with sweat chloride concentration below 60 mmol/L (within each trial) and below 30 mmol/L (within each trial) through week 24, and absolute change from baseline through week 52 in FEV_1_ % predicted and sweat chloride concentration, and safety and tolerability on the basis of adverse events, clinical laboratory values, ECGs, vital signs, and pulse oximetry.

### Statistical analysis

Efficacy was assessed in all participants with the intended *CFTR* genotype who were randomly assigned to treatment and received at least one dose of study drug during the treatment period (full analysis set). Estimand was defined for the primary and key secondary endpoints (appendix pp 20–21).

The primary endpoint was absolute change from baseline in FEV_1_ % predicted through week 24. The non-inferiority margin was selected on the basis of precedent in previous cystic fibrosis studies^18,19^ and is supported by a statistical approach using the Rothmann method. The Rothmann method recommends that the non-inferiority margin preserve at least 50% of the treatment effect of the active control (elexacaftor–tezacaftor–ivacaftor) compared with placebo, where the treatment effect is estimated by the lower bound of the 95% CI. The primary analysis of the primary estimand was performed using a mixed-effects model for repeated measures (MMRM) with change from baseline at day 15 and weeks 4, 8, 16, and 24 as the dependent variable. The model included fixed categorical effects for treatment, visit, age at screening (<18 years *vs* ≥18 years), and additionally genotype group for Trial VX20–121-103 (*F508del*-*F508del*, *F508del*-gating, *F508del*-residual function, and elexacaftor–tezacaftor–ivacaftor-responsive-non-*F508del*), and treatment-by-visit interaction, with baseline FEV_1_ % predicted and baseline sweat chloride as continuous covariates. The model was estimated using restricted maximum likelihood. Denominator degrees of freedom for the F-test for fixed effects were estimated using the Kenward-Roger approximation.^20^ An unstructured covariance structure was used to model the withinsubject errors. Missing data were assumed to be missing at random.

The estimated within-group least squares mean change from baseline and the least squares mean difference (vanzacaftor–tezacaftor–deutivacaftor *vs* elexacaftor–tezacaftor–ivacaftor) at each post-baseline visit were provided along with the corresponding two-sided 95% CI. The estimated within-group mean change and its SE at each post-baseline visit were plotted by treatment group. The primary result obtained from the model was the estimated treatment difference through week 24 (estimated by averaging least squares means at weeks 16 and 24 and taking the difference of vanzacaftor–tezacaftor–deutivacaftor compared with elexacaftor–tezacaftor–ivacaftor). The estimated treatment difference with a two-sided 95% CI and the one-sided p value for non-inferiority was provided. The primary null hypothesis was rejected, and non-inferiority demonstrated, if the lower bound of the 95% CI was –3·0 percentage points or greater.

Subgroup analyses of the primary endpoint were performed for age at screening, FEV_1_ % predicted at baseline, sex, sweat chloride at baseline, and geographical region using similar MMRM as described earlier. Additionally, a post-hoc subgroup analysis by genotype for Trial VX20–121-103 was done.

The analysis for the first key secondary efficacy endpoint was based on an MMRM similar to the primary analysis of the primary estimand, with absolute change from baseline in sweat chloride concentration at day 15, weeks 4, 16, and 24 as the dependent variable. Results from the MMRM were presented similarly to the results of the primary endpoint with the exception that two-sided p values were presented.

The response corresponding to sweat chloride concentration below 60 mmol/L (second key secondary endpoint) or below 30 mmol/L (third key secondary endpoint) at each visit through week 24 was analysed using a generalised estimating equations model. The model included fixed categorical effects for treatment, age at screening (<18 years *vs* ≥18 years), genotype group (*F508del*-minimal function, *F508del-F508del*, *F508del*-gating, *F508del*-residual function, and elexacaftor–tezacaftor–ivacaftor-responsive-non-*F508del*), visit, and treatment-by-visit interaction, with baseline FEV_1_ % predicted and baseline sweat chloride concen trations as continuous covariates. A logit link function and an unstructured working correlation matrix were used in the generalised estimating equations model. The estimated odds ratio (ORs) through week 24 (estimated by averaging weeks 16 and 24) along with the two-sided 95% CI and two-sided p value were presented. The estimates for each visit through week 24 were also presented. The number and proportion of participants with sweat chloride concentration below 60 mmol/L or below 30 mmol/L at each post-baseline visit through week 24 was descriptively summarised by treatment group. For the descriptive summary of results through week 24, the observed sweat chloride concentrations at weeks 16 and 24 were averaged and the value was used to determine the number and proportion of participants with sweat chloride concentration below 6 0 mmol/L or below 30 mmol/L.

A hierarchical testing procedure was used to control the overall type 1 error at an α of 0·05. The key secondary endpoints were formally tested at an α level of 0·05 only if the primary analysis of absolute change from baseline in FEV_1_ % predicted through week 24 was statistically significant. For a test at any step to be considered statistically significant within the testing hierarchy, it must have been statistically significant, and all previous tests (if any) within the hierarchy must have been statistically significant at the two-sided 0·05 level (one-sided 0·025 level for the primary endpoint). The testing order of the key secondary endpoints is provided in the appendix (p 22).

Trials VX20–121-102 and VX20–121-103 planned to enrol approximately 400 and 550 participants, respectively. For Trial VX20–121-102, assuming a within-group standard deviation of 8 and a 10% drop-out rate at week 24 and a treatment difference of 0 between vanzacaftor–tezacaftor–deutivacaftor and elexacaftor–tezacaftor–ivacaftor, a sample size of 200 participants in each group for a total of 400 participants would have more than 90% power to reject the null hypothesis that vanzacaftor–tezacaftor–deutivacaftor is inferior to elexacaftor–tezacaftor–ivacaftor (active control) by a margin that is greater than the non-inferiority margin of –3·0 percentage points in the absolute change from baseline in FEV_1_ % predicted through week 24, based on a one-sided, two-sample *t* test at a significance level of 0·025. Using the same assumptions for Trial VX20–121-103, a sample size of 275 participants in each group for a total of 550 participants would have more than 95% power to test the primary hypothesis for the primary endpoint.

For Trial VX20–121-102, for the key secondary efficacy endpoint of absolute change from baseline in sweat chloride concentration through week 24, assuming a within-group SD of 14 mmol/L and a 10% dropout rate at week 24, a sample size of 200 participants in each treatment group provided more than 90% power to detect a difference between the treatment groups of –5 mmol/L, based on a two-sided, two-sample *t* test at a significance level of 0·05. Using the same assumptions for Trial VX20–121-103, a sample size of 275 participants in each treatment group provided more than 95% power to detect a difference between the treatment groups.

For the key secondary efficacy endpoints of proportion of participants with sweat chloride concentrations below 60 mmol/L or below 30 mmol/L through week 24, participants from Trials VX20–121-102 and VX20–121-103 were pooled to provide sufficient power for analysis.

The secondary endpoint of number of pulmonary exacerbations through week 52 was analysed descriptively and the rate of pulmonary exacerbations per year was presented, along with difference in the rate between treatment groups and the associated 95% CI. A negative binomial distribution was assumed to obtain the two-sided 95% CI. Other secondary endpoints were analysed similarly to the primary or key secondary endpoints, as applicable (appendix pp 20–23).

For the primary endpoint and non-sweat chloride concentration-based endpoints, if the pre-dose day 1 value was missing, the most recent pre-dose, non-missing value on or after the day –14 visit, including unscheduled visits, was used as the baseline value. For sweat chloride concentration endpoints, for which baseline was the average of the two most recent pre-dose non-missing values on or after day –14, if only one non-missing value was available during this interval, the available value was considered to be the baseline value.

We did post-hoc subgroup analyses of the key secondary endpoint of absolute change in sweat chloride concentration by genotype for Trial VX20–121-103. We also did post-hoc subgroup analyses of this endpoint by sweat chloride concentration through week 24. Additionally, we did post-hoc analyses of the key secondary endpoints of proportion of participants with sweat chloride concentration below 60 mmol/L and below 30 mmol/L using shift tables to compare the proportions of participants above and below these thresholds; addi tional details are provided in the appendix (pp 23–25). Furthermore, we assessed duration of commercial elexacaftor–tezacaftor–ivacaftor use as previous medication as a post-hoc analysis (appendix p 24).

Based on a prespecified analysis plan, because of the similar population, study design and treatment duration, safety data from both trials were pooled for analysis. Safety data were descriptively summarised; no statistical testing on safety data was performed. Adverse events, including neuropsychiatric events, were collected during the trials and reported by the investigator as per the protocol. A broad neuropsychiatric events category (including 64 Preferred Terms per the Medical Dictionary for Regulatory Activities version 26.1) was created and included the following varied concepts: depression, suicide, anxiety, aggression, and insomnia. There were no formal mental health assessments conducted in Trials VX20–121-102 and VX20–121-103. In post-hoc analyses, we assessed the frequency of depression-related events. Additionally, in post-hoc analyses we assessed alanine aminotransferase and aspartate aminotransferase events and rash events through week 52 in the VX17–445-102 and VX17–445-105 trials,^5,21^ assessing elexacaftor–tezacaftor–ivacaftor (further details of post-hoc analyses are in the appendix [p 25]).We used SAS version 9.4 or higher to generate all statistical outputs. An independent data monitoring committee conducted planned safety reviews of trial data.

### Role of the funding source

The trial sponsor had a role in the design, data analysis, data interpretation, and writing of the report. The sponsor had no role in data collection; data collection was done by site investigators.

## Results

**Figure 1:**
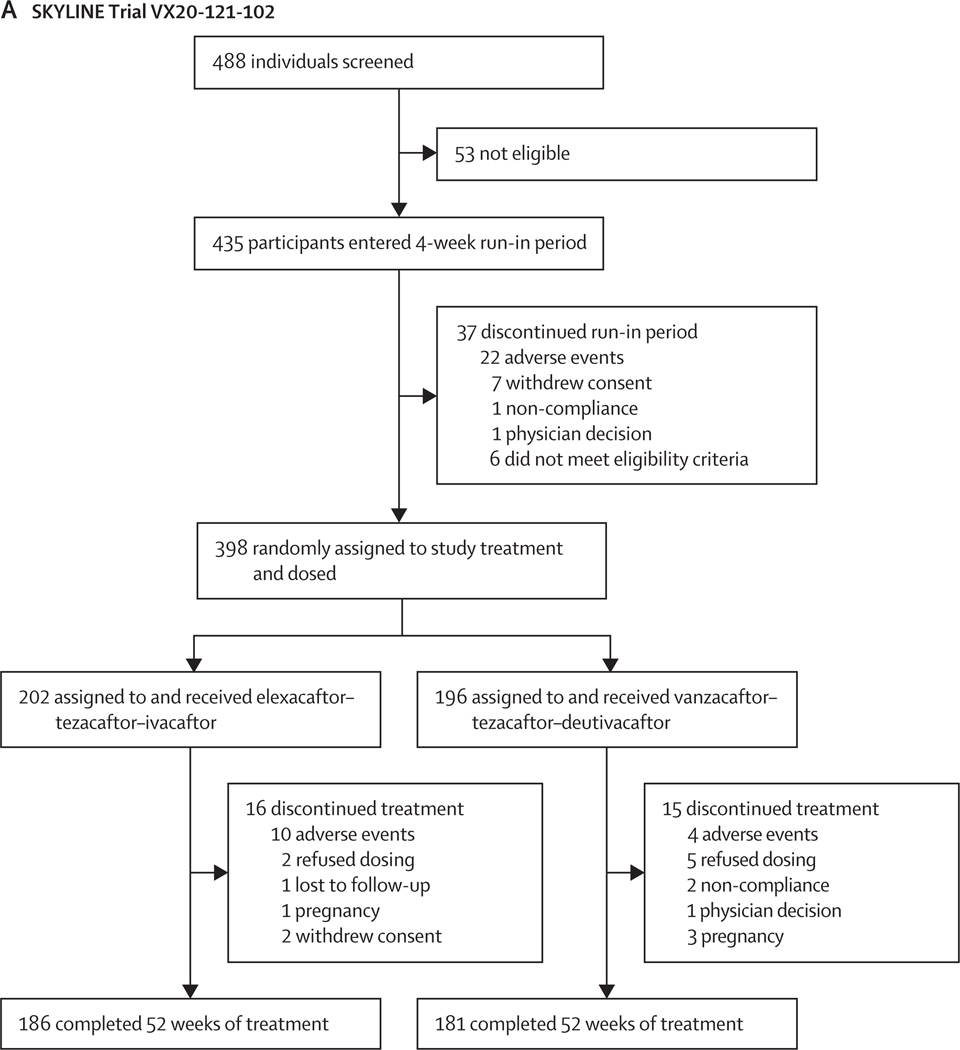

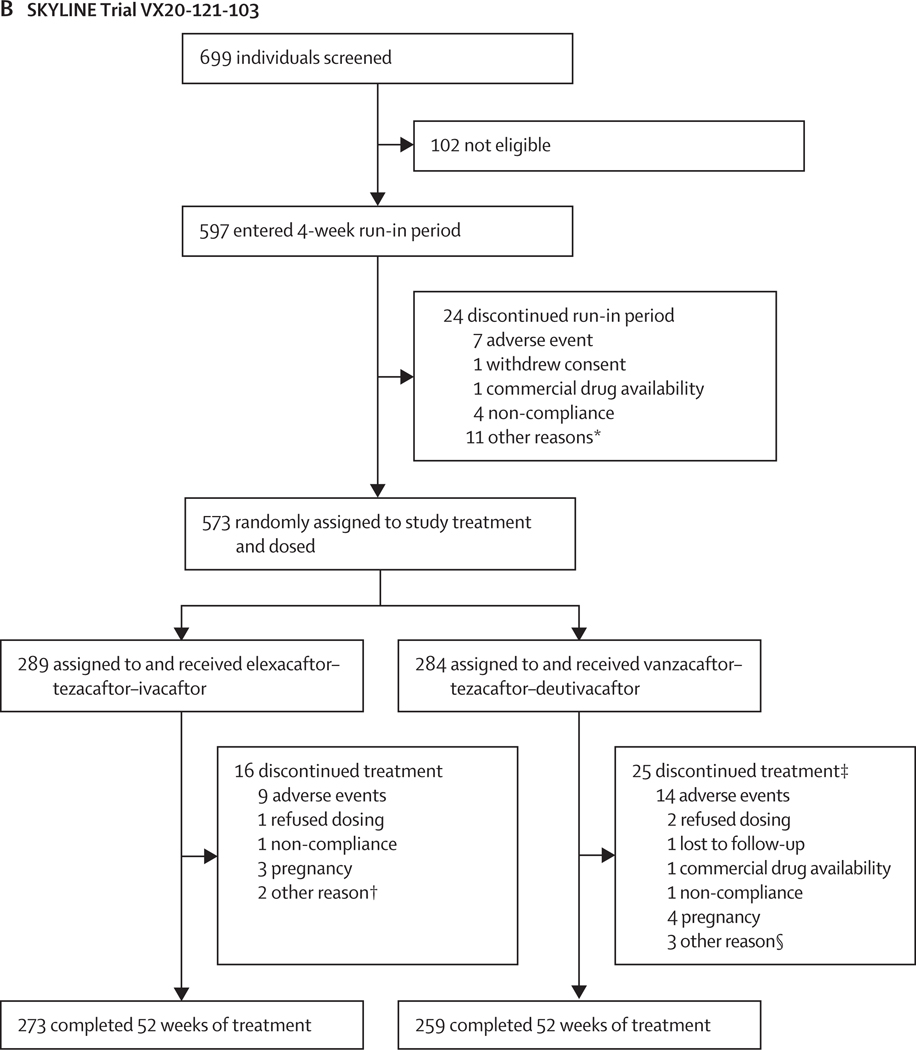
Trial profile for SKYLINE Trials VX20–121-102 (A) and VX20–121-103 (B) Some participants were taking elexacaftor–tezacaftor–ivacaftor before the run-in period. In Trials VX20–121-102 and VX20–121-103, three participants in the elexacaftor-tezacaftor-ivacaftor group and five participants in the vanzacaftor-tezacaftor-deutivacaftor group were randomly assigned to treatment but not dosed; these participants all discontinued the study during the run-in period. Commercial drug availability indicates that the participant chose to discontinue study treatment in favour of commercial drugs. *Other reasons for study discontinuation were: participant elected not to further participate in the study (n=3), refused dosing (n=1), did not meet eligibility (n=6), and run-in window expired (n=1). †Other reasons for treatment discontinuation were that participants wished to pursue pregnancy. ‡One participant had multiple reasons for discontinuation (adverse event and other reason). §Other reasons for treatment discontinuation were: participant wished to pursue pregnancy (n=1), withdrew consent (n=1), and partially quit dosing (n=1).

In SKYLINE Trial VX20–121-102, between Sept 14, 2021, and Oct 18, 2022, 488 individuals were screened, of whom 435 entered the 4-week run-in period and subsequently 37 discontinued the run-in period and were excluded from analysis. Hence, 398 participants were randomly assigned to either elexacaftor–tezacaftor–ivacaftor (n=202) or vanzacaftor–tezacaftor–deutivacaftor (n=196), received at least one dose of study drug, and had the intended *CTFR* genotype (full analysis set; figure 1A). Median age was 31·0 years (IQR 22·6–38·5), 163 (41%) of 398 participants were female, 235 (59%) were male, 388 (97%) were White, five (1%) were Black or African American, one (<1%) was other Asian, three (1%) were more than one race, and one (<1%) was other race (table 1).

In SKYLINE Trial VX20–121-103, between Oct 27, 2021, and Oct 26, 2022, 699 individuals were screened, of whom 597 entered the 4-week run-in period, and subsequently 24 discontinued the run-in period and were excluded from analysis. Hence, 573 participants were randomly assigned to either elexacaftor–tezacaftor–ivacaftor (n=289) or vanzacaftor–tezacaftor–deutivacaftor (n=284), received at least one dose of study drug, and had the intended *CTFR* genotypes (full analysis set; figure 1B). Median age was 33·1 years (IQR 24·5–42·2), 280 (49%) of 573 participants were female, 293 (51%) were male, 532 (93%) were White, three (1%) were more than one race, two (<1%) were other races, one (<1%) each was Southeast Asian, other Asian, and American Indian or Alaska Native, and 33 (6%) did not have race data collected due to local regulations (table 1). Additional baseline characteristics for both trials are in the appendix (p 41). Across both trials, 734 (76%) of 971 participants had previously received commercial elexacaftor–tezacaftor–ivacaftor, with a median exposure of approximately 2 years (post hoc; appendix p 42). Treatment groups in both trials were well matched at baseline (table 1; appendix p 41). After trial completion, 336 (84%) participants from Trial VX20–121-102 and 486 (85%) participants in Trial VX20–121-103 enrolled in an open-label study evaluating long-term treatment with vanzacaftor–tezacaftor–deutivacaftor.

**Figure 2:**
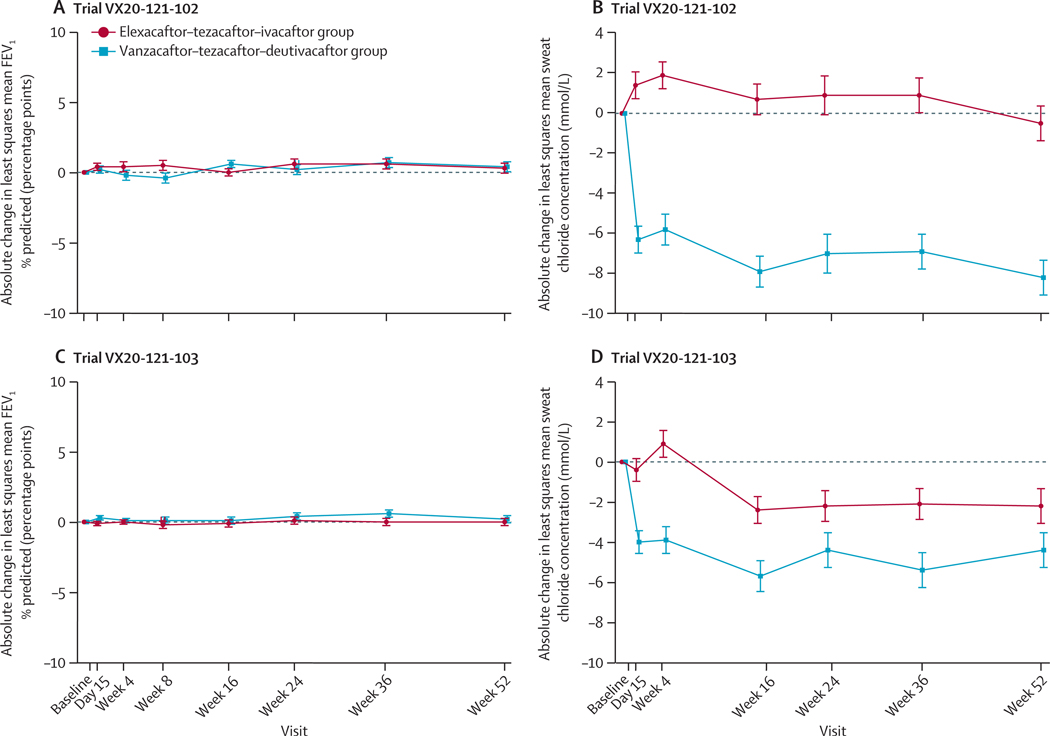
Absolute change in least squares mean from baseline in FEV_1_ % predicted and sweat chloride concentration FEV_1_ % predicted by visit for Trial VX20–121-102 (A) and Trial VX20–121–103 (C) and sweat chloride concentration by visit for Trial VX20–121-102 (B) and Trial VX20–121-103 (D). For FEV_1_ % predicted, baseline was the most recent non-missing measurement before the first dose of study drug on day 1. For sweat chloride concentration, baseline was defined as the average of the two most recent pre-dose, non-missing values on or after the day –14 visit, including unscheduled visits. Error bars show SEs.

In Trial VX20–121-102, the least squares mean absolute change in FEV_1_ % predicted from baseline through week 24 was 0·5 (SE 0·3) percentage points in the vanzacaftor–tezacaftor–deutivacaftor group versus 0·3 (0·3) percentage points in the elexacaftor–tezacaftor–ivacaftor group (least squares mean treatment difference of 0·2 percentage points [95% CI –0·7 to 1·1]; one-sided p<0·0001). In Trial VX20–121-103, the least squares mean absolute change in FEV_1_ % predicted from baseline through week 24 was 0·2 (SE 0·3) percentage points in the vanzacaftor–tezacaftor–deutivacaftor group versus 0·0 (0·2) percentage points in the elexacaftor–tezacaftor–ivacaftor group (least squares mean treatment difference of 0·2 percentage points [95% CI –0·5 to 0·9]; one-sided p<0·0001; table 2, figure 2A, C). Prespecified and post-hoc subgroup analyses were consistent with overall results for the primary endpoint for both trials (appendix p 29).

For the first key secondary endpoint of absolute change in sweat chloride concentration from baseline through week 24, the least squares mean treatment difference was –8·4 mmol/L (95% CI –10·5 to –6·3; two-sided p<0·0001) in Trial VX20–121-102 and –2·8 mmol/L (–4·7 to –0·9; two-sided p=0·0034) in Trial VX20–121-103 (table 2, figure 2B, D). Post-hoc genotype subgroup analyses in Trial VX20–121-103 were consistent with results for the first key secondary endpoint (appendix p 42). Post-hoc subgroup analysis for Trials VX20–121-102 and VX20–121-103 of absolute change in sweat chloride concentration are shown in the appendix (p 48). For the second and third key secondary endpoints, pooled analysis across both trials showed that a greater proportion of participants in the vanzacaftor–tezacaftor–deutivacaftor group had sweat chloride concentrations below 60 mmol/L through week 24 (399 [86%] of 465) than did those in the elexacaftor–tezacaftor–ivacaftor group (367 [77%] of 479; odds ratio 2·21 [95% CI 1·55 to 3·15]; p<0·0001]). Similarly, a greater proportion of participants across both trials in the vanzacaftor–tezacaftor–deutivacaftor group had sweat chloride con centrations below 30 mmol/L through week 24 (142 [31%] of 465) than did those in the elexacaftor–tezacaftor–ivacaftor group (108 [23%] of 479; odds ratio 2·87 [95% CI 2·00 to 4·12; p<0·0001; table 2). Post-hoc subgroup analyses of the proportion of participants with sweat chloride con centrations either above or below the thresholds of 30 mmol/L and 60 mmol/L at baseline compared to sweat chloride concentration through week 24 are shown in the appendix (pp 43–44).

The least squares mean treatment difference in CFQ-R respiratory domain score change from baseline through week 24 between the vanzacaftor–tezacaftor–deutivacaftor group and the elexacaftor–tezacaftor–ivacaftor group was 2·3 points (95% CI –0·6 to 5·2) in Trial VX20–121-102 and –0·1 points (–2·3 to 2·1) in Trial VX20–121-103 (appendix p 45). The annual rate of protocol-defined pulmonary exacerbation through week 52 was 0·32 in the vanzacaftor–tezacaftor–deutivacaftor group versus 0·42 in the elexacaftor–tezacaftor–ivacaftor group in Trial VX20–121-102 (treatment difference of –0·10 [95% CI –0·24 to 0·04]) and 0·29 versus 0·26 in Trial VX20–121-103 (treatment difference of 0·03 [95% CI –0·07 to 0·13]; appendix p 49). Results for absolute change from baseline in sweat chloride and FEV_1_ % predicted through week 52, and the proportion of participants (within trial) with sweat chloride concentrations below 60 mmol/L and below 30 mmol/L through week 24 are shown in the appendix (p 45).

Across both trials, 459 (96%) of 480 participants in the pooled vanzacaftor–tezacaftor–deutivacaftor group and 469 (96%) of 491 participants in the pooled elexacaftor–tezacaftor–ivacaftor group had at least one adverse event. Most adverse events were mild or moderate in severity and resolved without treatment interruption (table 3). The most common adverse events were infective pulmonary exacerbation (133 [28%] participants in the pooled vanzacaftor–tezacaftor–deutivacaftor group *vs* 158 [32%] in the pooled elexacaftor–tezacaftor–ivacaftor group), cough (108 [23%] *vs* 101 [21%]), COVID-19 (107 [22%] *vs* 127 [26%]), and nasopharyngitis (102 [21%] *vs* 95 [19%]). 68 (14%) of 480 participants in the pooled vanzacaftor–tezacaftor–deutivacaftor group and 81 (16%) of 491 in the pooled elexacaftor–tezacaftor–ivacaftor group had a serious adverse event, the most common of which was infective pulmonary exacerbation (29 [6%] *vs* 35 [7%]; appendix p 46). 18 (4%) participants in the pooled vanzacaftor–tezacaftor–deutivacaftor group and 18 (4%) in the pooled elexacaftor–tezacaftor–ivacaftor group discontinued treatment due to adverse events (table 3), the most common adverse events leading to treatment discontinuation were aminotransferase elevation events (appendix p 46). No deaths occurred during the treatment period. Two participants in the elexacaftor–tezacaftor–ivacaftor group in Trial VX20–121-103 died after the treatment period, both were assessed as not related to study treatment.

Based on laboratory values for alanine aminotransferase and aspartate aminotransferase, among 480 partici pants in the pooled vanzacaftor–tezacaftor–deutivacaftor group, 29 (6%), 12 (3%), and six (1%) had alanine aminotransferase or aspartate aminotransferase concentrations that were greater than three times, five times, and eight times the upper limit of normal (ULN), respectively, compared with 15 (3%), six (1%), and one (<1%) of 491 participants in the pooled elexacaftor–tezacaftor–ivacaftor group (appendix p 47). Differences in the occurrence of time-to-first aminotransferase elevations were observed in the first 3 months of the study treatment period and thereafter the occurrence of the events were similar between the treatment groups (appendix p 30). When analysing adverse events reported by investigators, there were 43 (9%) participants in the pooled vanzacaftor–tezacaftor–deutivacaftor group and 35 (7%) in the pooled elexacaftor–tezacaftor–ivacaftor group who had at least one aminotransferase elevation adverse event, and most of these events were mild or moderate in severity and resolved without treatment interruptio n (appendix p 47). Ten participants had aminotransferase elevation adverse events that led to treatment discontinuation: seven (1%) in the pooled vanzacaftor–tezacaftor–deutivacaftor group and three (1%) in the pooled elexacaftor–tezacaftor–ivacaftor group. Two (<1%) participants in the pooled vanzacaftor–tezacaftor–deutivacaftor group and two (<1%) in the pooled elexacaftor–tezacaftor–ivacaftor group had a serious aminotransferase elevation adverse event. There were no other clinically relevant differences between the two treatment groups in laboratory values. The incidences of aminotransferase elevations in both the pooled vanzacaftor–tezacaftor–deutivacaftor and elexacaftor–tezacaftor–ivacaftor groups in Trials VX20–121-102 and VX20–121-103 were lower than those observed in a post-hoc analysis of 52-week data from elexacaftor–tezacaftor–ivacaftor Trials VX17–445-102 and VX17–445-105 in participants who were CFTR modulator naive before receiving elexacaftor-tezacaftor-ivacaftor (appendix p 47).

53 (11%) of 480 participants in the pooled vanzacaftor–tezacaftor–deutivacaftor group and 38 (8%) of 491 in the pooled elexacaftor–tezacaftor–ivacaftor group had at least one rash event, and most were mild or moderate in severity (appendix p 48). Differences in the occurrence of time-to-first rash events were observed in the first month of study treatment, which then became similar between treatment groups thereafter (appendix p 30). One (<1%) participant in the pooled vanzacaftor–tezacaftor–deutivacaftor group had a rash event that led to treatment discontinuation within the first month of treatment.

The incidence of creatine kinase elevations was similar between treatment groups (appendix p 48). Overall, 43 (9%) participants in the pooled vanzacaftor–tezacaftor–deutivacaftor group and 41 (8%) in the pooled elexacaftor–tezacaftor–ivacaftor group had at least one creatine kinase elevation event. Most events were assessed by the investigator to be mild or moderate in severity and resolved without treatment interruption. Two participants had creatine kinase elevation events that led to treatment discontinuation, one (<1%) in the pooled vanzacaftor–tezacaftor–deutivacaftor group and one (<1%) in the pooled elexacaftor–tezacaftor–ivacaftor group. The elevated creatine kinase levels observed in these trials were generally asymptomatic and often associated with exercise.

55 (11%) of 480 participants in the pooled vanzacaftor–tezacaftor–deutivacaftor group and 59 (12%) of 491 in the pooled elexacaftor–tezacaftor–ivacaftor group had at least one neuropsychiatric event, and most were mild or moderate in severity (appendix p 49). Serious neuropsychiatric events occurred in four (1%) participants in the pooled vanzacaftor–tezacaftor–deutivacaftor group and four (1%) in the pooled elexacaftor–tezacaftor–ivacaftor group. Three (1%) participants in the pooled vanzacaftor–tezacaftor–deutivacaftor group and two (<1%) in the pooled elexacaftor–tezacaftor–ivacaftor group had neuropsychiatric events that led to treatment discon tinuation. Depression-related events, a subset of the neuropsychiatric events, occurred in 20 (4%) participants in the pooled vanzacaftor–tezacaftor–deutivacaftor group and 25 (5%) in the pooled elexacaftor–tezacaftor–ivacaftor group (appendix p 50).

We identified no clinically relevant changes in mean blood pressure (which remained in the normal range during the treatment period [appendix p 50]) or other vital signs, ECG, or pulse oximetry (data not shown).

## Discussion

We assessed the efficacy and safety of vanzacaftor–tezacaftor–deutivacaftor, a novel, once-daily CFTR modulator, compared with elexacaftor–tezacaftor–ivacaftor (standard of care for eligible people with cystic fibrosis) in 971 adolescents and adults with cystic fibrosis with diverse genotypes. Both trials met all primary and key secondary endpoints. Vanzacaftor–tezacaftor–deutivacaftor was non-inferior to elexacaftor–tezacaftor–ivacaftor in absolute change in FEV_1_ % predicted through week 24; elexacaftor–tezacaftor–ivacaftor was previously shown to have an approximately 14 percentage point increase in FEV_1_ % predicted compared with placebo in a phase 3 trial.^5^ Vanzacaftor–tezacaftor–deutivacaftor was superior to elexacaftor–tezacaftor–ivacaftor in terms of improvements in sweat chloride concentrations through week 24, with significant improvements in both trials. Treatment with vanzacaftor–tezacaftor–deutivacaftor led to 2·2 times greater odds of having sweat chloride concentrations below 60 mmol/L and 2·9 times greater odds of having sweat chloride concentrations below 30 mmol/L compared with elexacaftor–tezacaftor–ivacaftor. These results show that vanzacaftor–tezacaftor–deutivacaftor treatment might lead to more people with cystic fibrosis attaining levels of CFTR function either below the diagnostic threshold or in the normal range. Results of other secondary efficacy endpoints showed that treatment with vanzacaftor–tezacaftor–deutivacaftor led to maintenance of clinical benefit previously established with elexacaftor–tezacaftor–ivacaftor treatment. Vanzacaftor–tezacaftor–deutivacaftor was generally safe and well tolerated with a similar safety profile to elexacaftor–tezacaftor–ivacaftor. Additionally, vanzacaftor–tezacaftor–deutivacaftor is dosed once-daily, potentially reducing the medication burden for people with cystic fibrosis and facilitating adherence.^22–24^

Reflecting on the ability to improve CFTR function, in-vitro data from the FRT assay identified 31 additional *CFTR* variants that were responsive to vanzacaftor–tezacaftor–deutivacaftor but not responsive to elexacaftor-tezacaftor-ivacaftor. However, these in-vitro findings will need to be confirmed in real-world human studies to establish the clinical benefit in people with cystic fibrosis with these variants. Overall, vanzacaftor–tezacaftor–deutivacaftor had a similar effect on lung function as elexacaftor–tezacaftor–ivacaftor, with the potential to result in greater improvements in CFTR function in a broader population of people with cystic fibrosis.

Vanzacaftor–tezacaftor–deutivacaftor was non-inferior to elexacaftor–tezacaftor–ivacaftor in FEV_1_ % predicted. The trial was planned as a non-inferiority trial, because data from previous clinical trials suggested that further improvement in lung function (measured by FEV_1_ % predicted) beyond that provided by elexacaftor–tezacaftor–ivacaftor might not be possible in all participants due either to irreversible lung damage or relatively preserved lung function. For example, in previous phase 3 trials of elexacaftor–tezacaftor–ivacaftor, no further improvement in lung function in participants with *F508del*-*F508del* genotypes (who have two responsive variants) was seen compared with participants with *F508del*-minimal function genotypes (who have only one responsive variant), despite those with *F508del-F508del* genotypes having greater restoration of CFTR function, as measured by sweat chloride than those with *F508del*-minimal function genotypes.^2,5^ Although lung function remains a key clinical outcome and marker of disease progression in cystic fibrosis, improvement in sweat chloride con centration reflects restoration of the underlying dysfunction at the cause of cystic fibrosis. Sweat chloride is a direct, sensitive measure of CFTR function, is a well established diagnostic measure of cystic fibrosis, and predicts disease severity at a population level in natural history studies, although it has not been used to prospectively predict individual clinical benefit.^9,25,26^ Therefore, although new CFTR modulator therapies should demonstrate at least non-inferiority in terms of FEV_1_ % predicted improvement compared with standard of care, improvement in sweat chloride concentration allows an opportunity to differentiate between CFTR modulator regimens. This evolution from measurement of important, but less sensitive, clinical measures (eg, FEV_1_ % predicted) to other more sensitive measures reflecting the underlying pathophysiology (eg, sweat chloride) has precedent in other disease areas, such as HIV, cancer, and hepatitis C virus infection.^27–29^ For these diseases, as more effective medicines were developed, more sensitive endpoints have been used to differentiate between treatments.

In the trials presented here, significant improvements in all the key secondary endpoints related to sweat chloride concentrations were observed through 24 weeks with vanzacaftor–tezacaftor–deutivacaftor treatment compared with elexacaftor–tezacaftor–ivacaftor. The magnitude of the absolute change in sweat chloride concentration in the Trial VX20–121-102 population (*F508del*-minimal function genotypes) was greater than in the Trial VX20–121-103 population (*F508del*-*F508del, F508del-*gating, *F508del-*residual function, and elexacaftor–tezacaftor–ivacaftor-responsive-non*-F508del* genotypes), possibly because participants in Trial VX20–121-102 had a higher mean sweat chloride at baseline than did those in Trial VX20–121-103. Overall, the improvements in sweat chloride concentrations seen in both trials address the causal biology of cystic fibrosis and advance the field towards the goal of reaching normal levels of CFTR function seen in people without cystic fibrosis.

The clinical relevance of the sweat chloride concentration thresholds of 60 mmol/L (above which the diagnosis of cystic fibrosis is likely) and 30 mmol/L (below which cystic fibrosis is not diagnosed, and which reflects normal levels) is supported by natural history and clinical trial data.^10,14,26^ The statistically superior improvements in sweat chloride concentrations through 24 weeks of vanzacaftor–tezacaftor–deutivacaftor treatment compared with elexacaftor–tezacaftor–ivacaftor mean that more people with cystic fibrosis might reach these thresholds. Although additional long-term data are needed to determine whether these thresholds for diagnosis and prognosis of cystic fibrosis are clinically relevant to predict long-term treatment response, further improve ment in CFTR function (as measured by improve ments in sweat chloride concentrations) to normal levels remains a goal of therapy. Correction of CFTR function to levels seen in individuals without cystic fibrosis early in life has the best potential to restore normal physiology and prevent disease development or progression, or both.^30^

Vanzacaftor–tezacaftor–deutivacaftor was generally safe and well tolerated. Because of the active comparator, the studies excluded participants who had a history of intolerance to elexacaftor–tezacaftor–ivacaftor and all participants received and had to tolerate elexacaftor–tezacaftor–ivacaftor during the 4-week run-in period. Hence, the safety of elexacaftor–tezacaftor–ivacaftor in these trials reflected the experience of participants who had previously received and tolerated elexacaftor–tezacaftor–ivacaftor, whereas the safety in the vanzacaftor–tezacaftor–deutivacaftor group reflected that of par ticipants who had started a new CFTR modulator. The most common adverse events and serious adverse events were generally consistent with common manifestations in cystic fibrosis. The occurrence of serious adverse events and events that led to treatment discontinuation were low and similar between groups. Importantly, in post-hoc analyses, we found that 76% of participants previously received commercial elexacaftor–tezacaftor–ivacaftor, with a median exposure of approximately 2 years and all participants received elexacaftor–tezacaftor–ivacaftor during the run-in period. This is relevant when interpreting rates of aminotransferase elevations and rash events (which are known to occur early after elexacaftor–tezacaftor–ivacaftor initiation^5^) because these events would have already occurred before entering the treatment period of Trials VX20–121-102 and VX20–121-103. This hypothesis is supported by the incidence of alanine aminotransferase or aspartate aminotransferase elevations that were greater than three times, five times, and eight times the ULN in the pooled elexacaftor–tezacaftor–ivacaftor group being lower than those previously seen in the elexacaftor–tezacaftor–ivacaftor group in the phase 3 Trial VX17–445-102,^5^ in which participants were naive to CFTR modulator treatment (incidences of alanine aminotransferase or aspartate aminotransferase elevations that were more than three times, five times, and eight times the ULN were 7·9%, 2·5%, and 1·5%, respectively).^5^ The incidence of rash events in the pooled elexacaftor–tezacaftor–ivacaftor group was also lower than in the elexacaftor–tezacaftor–ivacaftor group in Trial VX17–445-102 (8% *vs* 11%). Furthermore, the incidence of elevated aminotransferase and rash events in both the pooled vanzacaftor–tezacaftor–deutivacaftor group and the pooled elexacaftor–tezacaftor–ivacaftor group is lower than that observed in the 52-week experience with elexacaftor–tezacaftor–ivacaftor treatment in Trials VX17–445-102 and open-label extension VX17–445-105 in CFTR-modulator naive participants. Additionally, the known early time course of drugrelated liver and rash events after the initiation of a drug explains why the incidence of first aminotransferase elevation or first rash event were higher in the vanzacaftor–tezacaftor–deutivacaftor group in the first month (rash) and 3 months (aminotransferase elevations) of treatment than in the elexacaftor–tezacaftor–ivacaftor group, and the incidence was similar thereafter.

The high burden of mental health conditions in people with cystic fibrosis, including depression, anxiety, suicidal ideation, insomnia, and other conditions, is well established in the published literature.^31–34^ Previous analysis of pooled placebo-controlled elexacaftor–tezacaftor–ivacaftor clinical trials has shown that the inci dence of depression-related events was similar between the elexacaftor–tezacaftor–ivacaftor group (3·32 events per 100 person years) and the placebo group (3·24 events per 100 person years).^35^ Results from Trials VX20–121-102 and VX20–121-103 showed that the incidence of neuropsychiatric events reported by the investigators in these trials was similar between treatment groups and was consistent with the background rate of these events in people with cystic fibrosis not receiving CFTR modulator therapy. The cumulative review of the elexacaftor–tezacaftor–ivacaftor data, including from clinical trials, post-marketing reports, an ongoing registry-based post authorisation safety study, and peer-reviewed literature, suggests that depression symptoms and depression-related events reported in people with cystic fibrosis treated with elexacaftor–tezacaftor–ivacaftor are gen erally consistent with background epidemiology of these events in the cystic fibrosis population and do not suggest a causal relationship with CFTR modulator treatment.

To complement the clinical trial results, in-vitro experiments using the FRT system were conducted to identify additional *CFTR* variants responsive to vanzacaftor–tezacaftor–deutivacaftor. The in-vitro FRT assay is clinically validated, has been shown to be highly predictive of clinical benefit, and has been accepted by regulatory authorities for previous CFTR modulators.^36–38^ In-vitro testing confirmed that all previously identified elexacaftor–tezacaftor–ivacaftor-responsive variants were also responsive to vanzacaftor–tezacaftor–deutivacaftor. Furthermore, reflecting the increased efficacy of vanzacaftor–tezacaftor–deutivacaftor, the FRT assay identified 31 *CFTR* variants that were responsive to vanzacaftor–tezacaftor–deutivacaftor but not responsive to elexacaftor–tezacaftor–ivacaftor (and are not currently approved for treatment with any CFTR modulator therapy). Notably, although a lack of responsiveness in the FRT system does not predict lack of clinical benefit (eg, a *CFTR* variant that does not meet the threshold for in-vitro responsiveness might still be clinically responsive), these data suggest that vanzacaftor–tezacaftor–deutivacaftor has the potential to treat a broader population of people with cystic fibrosis com pared with elexacaftor–tezacaftor–ivacaftor through an expanded indication.

There is no longer clinical equipoise to conduct a placebo-controlled trial in participants with responsive *CFTR* variants due to the availability of elexacaftor–tezacaftor–ivacaftor. Although elexacaftor–tezacaftor–ivacaftor is the most appropriate active comparator for a clinical trial, one limitation of its use is that efficacy and safety are evaluated relative to elexacaftor–tezacaftor–ivacaftor, rather than to placebo. Additionally, because most participants had previously received elexacaftor–tezacaftor–ivacaftor, a controlled trial with an elexacaftor–tezacaftor–ivacaftor run-in period means that adverse event data are reflective only of participants who are able to tolerate elexacaftor–tezacaftor–ivacaftor. Therefore, the trial design does not allow the assessment of the efficacy and safety of vanzacaftor–tezacaftor–deutivacaftor in people either unable to tolerate elexacaftor–tezacaftor–ivacaftor or naive to elexacaftor–tezacaftor–ivacaftor. There might be limitations in detecting further improvements in lung function as measured by spirometry in clinical trials. Other assessments besides spirometry (FEV_1_ % predicted) might be able to detect modest changes in lung function, including lung clearance index (which was an endpoint in the vanzacaftor–tezacaftor–deutivacaftor paediatric trial^17^); however, there are technical challenges in performing lung clearance index assessments in older people with cystic fibrosis, who typically have more severe airway obstruction.^39,40^ Additionally, our trials are limited by the lack of ethnic and racial diversity of the population, with more than 90% of participants being White and not Latino or Hispanic, which might limit the generalisability of our findings. Hence, future trials must make efforts to enhance clinical trial participation to generate data in a broader and more representative ethnic and racial distribution that better reflects the cystic fibrosis population.

In summary, these two large, international phase 3 trials show that vanzacaftor–tezacaftor–deutivacaftor, a novel, once-daily, triple combination CFTR modulator therapy, was non-inferior in terms of change from baseline in lung function and superior in improvements in sweat chloride concentrations compared with the current standard of care, elexacaftor–tezacaftor–ivacaftor, in eligible people with cystic fibrosis. Results of in-vitro analyses indicate 31 additional *CFTR* variants responsive to vanzacaftor–tezacaftor–deutivacaftor but not to elexacaftor–tezacaftor–ivacaftor, potentially expanding eligibility to CFTR modulator treatment. Vanzacaftor–tezacaftor–deutivacaftor is a CFTR modulator that provides more convenient once-daily dosing, is as efficacious in improving lung function compared to elexacaftor–tezacaftor–ivacaftor with further restoration of CFTR function, and has the potential to treat a broader population than the current standard of care CFTR modulators.

## Supplementary Material

1

## Figures and Tables

**Table 1:** Baseline demographic and clinical characteristics, full analysis set

	Trial VX20-121-102		Trial VX20-121-103	
	Elexacaftor-tezacaftor-ivacaftor group (N=202)	Vanzacaftor-tezacaftor-deutivacaftor group (N=196)	Elexacaftor-tezacaftor-ivacaftor group (N=289)	Vanzacaftor-tezacaftor-deutivacaftor (N=284)
Sex				
Female	83 (41%)	80 (41%)	145 (50%)	135 (48%)
Male	119 (59%)	116 (59%)	144 (50%)	149 (52%)
Age, years	31·3 (21·9–38·5)	30·3 (22·8–37·5)	33·8 (24·7–43·1)	32·6 (24·1–41·5)
≥12 to <18	31 (15%)	26 (13%)	38 (13%)	41 (14%)
≥18	171 (85%)	170 (87%)	251 (87%)	243 (86%)
Ethnicity				
Hispanic or Latino	11 (5%)	13 (7%)	5 (2%)	4 (1%)
Not Latino or Hispanic	190 (94%)	183 (93%)	261 (90%)	265 (93%)
Not collected per local Regulations	1 (<1%)	0	23 (8%)	15 (5%)
Race				
White	197 (98%)	191 (97%)	262 (91%)	270 (95%)
Black or African American	1 (<1%)	4 (2%)	0	0
Southeast Asian	0	0	0	1 (<1%)
Other Asian	0	1 (<1%)	1 (<1%)	0
American Indian or Alaska Native	0	0	1 (<1%)	0
Other race	1 (<1%)	0	1 (<1%)	1 (<1%)
Not collected per local Regulation	0	0	23 (8%)	10 (4%)
More than one race	3 (1%)	0	1 (<1%)	2 (1%)
FEV_1_ % predicted, percentage points	67·2 (14·6)	67·0 (15·3)	66·4 (14·9)	67·2 (14·6)
Sweat chloride concentration, mmol/L	54·3 (18·2)	53·6 (17·0)	42·1 (17·9)	43·4 (18·5)
≥30 to <60	105 (52%)	114 (58%)	154 (53%)	158 (56%)
<30	19 (9%)	17 (9%)	80 (28%)	72 (25%)
CFQ-R respiratory domain score	82·9 (15·7)	85·8 (14·7)	85·6 (13·2)	85·7 (13·2)
BMI, kg/m²	23·0 (3·9)	22·7 (3·4)	22·9 (3·3)	23·3 (4·0)
Previous CFTR modulator use				
Any	177 (88%)	170 (87%)	250 (87%)	241 (85%)
Elexacaftor–tezacaftor–ivacaftor	177 (88%)	168 (86%)	204 (71%)	185 (65%)
Genotype group				
*F508del-F508del*	NA	NA	224 (78%)	222 (78%)
*F508del-*gating	NA	NA	20 (7%)	19 (7%)
*F508del-*residual Function	NA	NA	23 (8%)	23 (8%)
Elexacaftor-tezacaftor-ivacaftor-responsive non-*F508del*	NA	NA	22 (8%)	20 (7%)
*F508del-*minimal Function	202 (100%)	196 (100%)	NA	NA

Data are n (%), median (IQR), or mean (SD). CFQ-R=Cystic Fibrosis Questionnaire–Revised. NA=not applicable.

**Table 2:** Primary and key secondary efficacy endpoints in Trials VX20–121–102 and VX20–121–103, full analysis set

	Trial VX20-121-102		Trial VX20-121-103		Pooled analyses	
	Elexacaftor-tezacaftor-ivacaftor group (N=202)	Vanzacaftor-tezacaftor-deutivacaftor group (N=196)	Elexacaftor-tezacaftor-ivacaftor group (N=289)	Vanzacaftor-tezacaftor-deutivacaftor (N=284)	Elexacaftor-tezacaftor-ivacaftor group (N=491)	Vanzacaftor-tezacaftor-deutivacaftor group (N=480)
**Primary endpoint**						
Absolute change in FEV_1_ % predicted from baseline through week 24,* percentage points						
Baseline, mean (SD)	67·2 (14·6)	67·0 (15·3)	66·4 (14·9)	67·2 (14·6)	NA	NA
Absolute change, least squares mean (SE; 95% CI)	0·3 (0·3; −0·3 to 0·9)	0·5 (0·3; −0·1 to 1·1)	0·0 (0·2; −0·5 to 0·5)	0·2 (0·3; −0·3 to 0·7)	NA	NA
Least squares mean difference *vs* elexacaftor–tezacaftor–ivacaftor group (95% CI)	··	0·2 (−0·7 to 1·1)	··	0·2 (−0·5 to 0·9)	NA	NA
One-sided p_non-inferiority_	··	<0·0001	··	<0·0001	NA	NA
**Key secondary endpoints**						
Absolute change in sweat chloride concentration from baseline through week 24*, mmol/L						
Baseline, mean (SD)	54·3 (18·2)	53·6 (17·0)	42·1 (17·9)	43·4 (18·5)	NA	NA
Absolute change, least squares mean (SE; 95% CI)	0·9 (0·8; −0·6 to 2·3)	−7·5 (0·8; −9·0 to −6·0)	−2·3 (0·7; −3·6 to −0·9)	−5·1 (0·7; −6·4 to −3·7)	NA	NA
Least squares mean difference *vs* elexacaftor–tezacaftor–ivacaftor group (95% CI)	··	−8·4 (−10·5 to −6·3)	··	−2·8 (−4·7 to −0·9)	NA	NA
Two-sided p_superiority_	··	<0·0001	··	0·0034	NA	NA
Proportion of participants with sweat chloride concentration <60 mmol/L through week 24*						
Baseline	NA	NA	NA	NA	358/483 (74%)	361/476 (76%)
Week 24	NA	NA	NA	NA	367/479 (77%)	399/465 (86%)
Odds ratio† *vs* elexacaftor–tezacaftor–ivacaftor group (95% CI)	NA	NA	NA	NA	··	2·21 (1·55 to 3·15)
Two-sided p_superiority_	NA	NA	NA	NA	··	<0·0001
Proportion of participants with sweat chloride concentration <30 mmol/L through week 24*						
Baseline	NA	NA	NA	NA	99/483 (21%)	89/476 (19%)
Week 24	NA	NA	NA	NA	108/479 (23%)	142/465 (31%)
Odds ratio† *vs* elexacaftor–tezacaftor–ivacaftor group (95% CI)	NA	NA	NA	NA	··	2·87 (2·00 to 4·12)
Two-sided p_superiority_	NA	NA	NA	NA	··	<0·0001

Except for sweat chloride, baseline was defined as the pre-dose day 1 value. For sweat chloride, baseline was defined as the average of the 2 most recent pre-dose, non-missing values on or after the day –14 visit, including unscheduled visits. NA=not applicable.

* Estimates through week 24 were obtained by averaging estimates at weeks 16 and 24.

† The generalised estimating equation model was used to estimate the odds ratio; observed proportion is presented.

**Table 3:** Adverse events in the treatment period, in pooled Trials VX20-121-102 and VX20-121-103

	Pooled elexacaftor-tezacaftor-ivacaftor group (N=491)	Pooled vanzacaftor-tezacaftor-deutivacaftor group (N=480)
Participants with any adverse event	469 (96%)	459 (96%)
Maximum severity of adverse event		
Mild	145 (30%)	166 (35%)
Moderate	269 (55%)	239 (50%)
Severe	54 (11%)	54 (11%)
Life-threatening	1 (<1%)	0
Participants with adverse events leading to discontinuation of trial regimen*	18 (4%)	18 (4%)
Laboratory values†	6 (1%)	9 (2%)
Psychiatric disorders	2 (<1%)	3 (1%)
Skin and subcutaneous tissue disorders	0	1 (<1%)
Other	10 (2%)	5 (1%)
Participants with adverse events leading to interruption of trial regimen	12 (2%)	20 (4%)
Participants with serious adverse events	81 (16%)	68 (14%)
Participants with adverse events leading to death	0‡	0
Participants with adverse events that occurred in ≥10% of participants in any treatment group		
Infective pulmonary exacerbation of cystic fibrosis	158 (32%)	133 (28%)
Cough	101 (21%)	108 (23%)
COVID-19	127 (26%)	107 (22%)
Nasopharyngitis	95 (19%)	102 (21%)
Headache	63 (13%)	76 (16%)
URTI	67 (14%)	72 (15%)
Oropharyngeal pain	60 (12%)	69 (14%)
Diarrhoea	59 (12%)	58 (12%)
Influenza	26 (5%)	52 (11%)
Pyrexia	50 (10%)	52 (11%)
Fatigue	46 (9%)	51 (11%)
Nasal congestion	47 (10%)	48 (10%)
Increased sputum	50 (10%)	45 (9%)

Data are n (%) where n is participants. URTI=upper respiratory tract infection.

* Adverse events that led to discontinuation in at least two participants, according to preferred terms, are listed in the appendix (p 46).

† Adverse events that occurred in at least two participants in either group included increased alanine aminotransferase, aspartate aminotransferase, increased blood bilirubin, increased blood bilirubin unconjugated, and blood alkaline phosphatase.

‡ Two participants in the elexacaftor–tezacaftor–ivacaftor group in Trial VX20–121-103 died after the treatment period, both were assessed as not related to study treatment.

## Data Availability

Vertex Pharmaceuticals is committed to advancing medical science and improving patient health. This includes the responsible sharing of clinical trial data with qualified researchers. Proposals for the use of these data will be reviewed by a scientific board. Approvals are at the discretion of Vertex and will be dependent on the nature of the request, the merit of the research proposed, and the intended use of the data. Please contact CTDS@vrtx.com if you would like to submit a proposal or need more information.

## Research in context

### Evidence before this study

We searched PubMed for articles published in English from database inception to Oct 10, 2024, using the terms “CFTR modulator”, “clinical trial”, “vanzacaftor”, and “elexacaftor”. Previous research efforts in CFTR modulator therapeutics led to development of elexacaftor–tezacaftor–ivacaftor (the current standard of care for people with cystic fibrosis who are eligible, tolerate the drug, and have access), which has provided remarkable improvement in lung function and other clinical outcomes for many people with cystic fibrosis, alongside delivering substantial reductions in sweat chloride—a sensitive in-vivo test of CFTR function. However, potential exists for further improvement of CFTR function, addressing the underlying cause of cystic fibrosis. Phase 2 trials of vanzacaftor–tezacaftor–deutivacaftor found this triple combination therapy to be generally safe and well tolerated and showed a greater reduction in sweat chloride concentrations and improvement in lung function relative to baseline when compared with tezacaftor–ivacaftor in participants with an *F508del*-*F508del* homozygous genotype

### Added value of this study

We report results from two large phase 3 clinical trials designed to assess the novel, once-daily triple combination modulator vanzacaftor–tezacaftor–deutivacaftor versus elexacaftor–tezacaftor–ivacaftor in individuals aged 12 years and older with cystic fibrosis and a broad range of genotypes over 52 weeks. After establishing a stable baseline on elexacaftor–tezacaftor–ivacaftor, treatment with vanzacaftor–tezacaftor–deutivacaftor showed non-inferiority with regards to change in lung function as measured by FEV_1_ % predicted and resulted in greater improvement in sweat chloride concentrations versus participants treated with elexacaftor–tezacaftor–ivacaftor as an active control.

### Implications of all the available evidence

These studies show that once-daily vanzacaftor-tezacaftor-deutivacaftor is safe and as efficacious as elexacaftortezacaftor-ivacaftor in individuals aged 12 years and older with cystic fibrosis with regards to lung function (as measured by spirometry), pulmonary exacerbations, and other clinical outcomes. Vanzacaftor–tezacaftor–deutivacaftor provided significant improvements in sweat chloride concentrations compared with elexacaftor–tezacaftor–ivacaftor. These two trials in adolescents and adults, along with the paediatric study in children with cystic fibrosis aged 6–11 years (RIDGELINE Trial VX21–121-105), comprise the most comprehensive pivotal programme conducted in cystic fibrosis, with more than 1000 participants. These results demonstrate the potential for vanzacaftor–tezacaftor–deutivacaftor to further improve the lives of people with cystic fibrosis by limiting disease manifestation in those who are younger and preventing progression of disease in adults. Vanzacaftor–tezacaftor–deutivacaftor may have the potential to further restore CFTR function in a broader population compared with elexacaftor-tezacaftor-ivacaftor; however, these in-vitro findings will need to be confirmed in real-world studies to establish benefits in people with cystic fibrosis with these variants.
