# Supplementary material for: Vanzacaftor–tezacaftor–deutivacaftor versus elexacaftor–tezacaftor–ivacaftor in individuals with cystic fibrosis aged 12 years and older (SKYLINE Trials VX20–121-102 and VX20–121-103): results from two randomised, active-controlled, phase 3 trials
Source: Lancet Respir Med. Author manuscript; Available in PMC 2025 Jun 23. (PMC12184100; doi:10.1016/S2213-2600(24)00411-9)
Supplement: 1 [file NIHMS2081037-supplement-1.pdf]

THE LANCET
Respiratory Medicine

Supplementary appendix

This appendix formed part of the original submission and has been peer reviewed. We post it as supplied by the authors.

Supplement to: Keating C, Yonker LM, Vermeulen F, et al. Vanzacaftor–tezacaftor–deutivacaftor versus elexacaftor–tezacaftor–ivacaftor in individuals with cystic fibrosis aged 12 years and older (SKYLINE Trials VX20-121-102 and VX20-121-103): results from two randomised, active-controlled, phase 3 trials. *Lancet Respir Med* 2025; published online Jan 2. https://doi.org/10.1016/S2213-2600(24)00411-9.

Appendix to:

Vanzacaftor-tezacaftor-deutivacaftor versus elexacaftor-tezacaftor-ivacaftor in individuals with cystic fibrosis aged 12 years and older (SKYLINE Trials VX20-121-102 and VX20-121-103): results from two randomised, active-controlled, phase 3 trials

Claire Keating MD,[1] Lael M. Yonker, MD,[2] François Vermeulen MD,[3] Dario Prais MD,[4] Rachel W. Linnemann MD,[5] Aaron Trimble MD,[6] Tom Kotsimbos, MD[7] Joel Mermis MD,[8] Andrew T. Braun MD,[9] Mark O'Carroll MBChB,[10] Sivagurunathan Sutharsan MD,[11] Prof Emeritus Bonnie Ramsey MD,[12] Prof Marcus A. Mall MD,[13 a,b,c] Prof Jennifer L. Taylor-Cousar MD,[14] Edward F. McKone MD,[15] Prof Elizabeth Tullis MD,[16] Tim Floreth, MD,[17] Peter Michelson MD,[17] Patrick R. Sosnay MD,[17] Nitin Nair PhD,[17] Rachel Zahigian PhD,[17] Hannah Martin MD,[17] Neil Ahluwalia MD,[17] Anna Lam MD,[17] Prof Alexander Horsley PhD[18]

[1]Columbia University Irving Medical Center, New York, NY, US (C Keating, MD); [2]Massachusetts General Hospital, Boston, MA, US (L M Yonker, MD); [3]Cystic Fibrosis Reference Centre, Department of Pediatrics, Katholieke Universiteit Leuven, Leuven, Belgium (F Vermeulen, MD); [4] Pediatric Pulmonology Institute, Schneider Children's Medical Center and Faculty of Medical and Health Sciences, Tel Aviv University, Tel Aviv, Israel (D Prais, MD); [5]Emory University, Children's Healthcare of Atlanta, Atlanta, GA, US (R W Linnemann, MD); [6]Oregon Health and Science University, Portland, OR, US (A Trimble, MD); [7]Alfred Hospital, Monash University Melbourne, Melbourne, Australia (T Kotsimbos, MD); [8]University of Kansas Medical Center, Kansas City, KS, US (J Mermis, MD); [9]Department of Medicine, Division of Pulmonary and Critical Care Medicine, University of Wisconsin School of Medicine and Public Health, Madison, WI, US (A T Braun, MD); [10]Auckland City Hospital, Health New Zealand, Auckland, New Zealand (M O'Carroll, MBChB); [11]Department of Pulmonary Medicine, Division of Cystic Fibrosis, University Medicine Essen-Ruhrlandklinik, University of Duisburg-Essen, Essen, Germany (S Sutharsan, MD); [12]Seattle Children's Hospital, University of Washington, Seattle, WA, US (B Ramsey, MD); [13a] Department of Pediatric Respiratory Medicine, Immunology and Critical Care Medicine, Charité - Universitätsmedizin Berlin, Berlin, Germany (M A Mall, MD); [13b]German Center for Lung Research (DZL), associated partner site Berlin, Berlin, Germany (M A Mall, MD); [13c]German Center for Child and Adolescent Health (DZKJ), partner site, Berlin, Germany (M A Mall, MD);[14]National Jewish Health, Denver, CO, US (J L Taylor-Cousar, MD); [15]St. Vincent's University Hospital, University College Dublin, Dublin, Ireland (E McKone, MD); [16]St Michael's Hospital, University of Toronto, Toronto, ON, Canada (E Tullis, MD); [17]Vertex Pharmaceuticals, Boston, MA, US (T Floreth, MD; P Michelson, MD; P R Sosnay, MD; N Nair, PhD; R Zahigian, PhD; H Martin, MD; N Ahluwalia, MD; A Lam, MD); [18]Divsion of Infection, Immunity and Respiratory Medicine, University of Manchester, Manchester, UK (A Horsley, PhD).

Table of Contents

List of Site Investigators 4
Supplementary Methods 10
In Vitro Assay of CFTR Function 10
Trials VX20-121-102 and VX20-121-103 Eligibility Criteria 11
Schedule of Assessments (Trials VX20-121-102 and VX20-121-103) 19
Statistical Analysis 20
Adverse Event Definition and Reporting 25
Protocol Deviations 26
Supplementary Results 27
Supplementary Figures 29
Figure S1. Subgroup Analysis of Absolute Change in $ppFEV_1$ From Baseline Through Week 24. 29
Figure S2. Time-to-First ALT/AST >3×ULN Event During the Treatment Emergent Period for the Treatment Period 30
Figure S3. Time-to-First Rash Event During the Treatment Emergent Period for Treatment Period 30
Supplementary Tables 31
Table S1. Eligible Minimal Function *CFTR* Variants for Trial VX20-121-102.* 31
Table S2. Trial VX20-121-102 Participant Enrollment by Country and Site 32
Table S3. Trial VX20-121-103 Participant Enrollment by Country and Site 35
Table S4. Trial VX20-121-102 List of IECs/ and IRBs and Approvals 39
Table S5. Trial VX20-121-103 List of IECs/ and IRBs and Approvals 40
Table S6. Additional Baseline Characteristics.* 41
Table S7. Post-hoc Duration of ELX/TEZ/IVA Use as a Prior Medication (Pooled From Trials VX20-121-102 and VX20-121-103). 42
Table S8. *CFTR* Variants Demonstrated to be Responsive Only to VNZ/TEZ/D-IVA in the FRT Assay. 42
Table S9. Post hoc Absolute Change in Sweat Chloride Concentration From Baseline Through Week 24 By Genotype in Trial VX20-121-103 42
Table S10. Post hoc Shift Tables for Proportion of Participants With Sweat Chloride <60 mmol/L Through Week 24 (Pooled from Trials VX20-121-102 and VX20-121-103) 43
Table S11. Post hoc Subgroup Analysis of Absolute Change From Baseline in Sweat Chloride (mmol/L) Through Week 24 for Participants With Sweat Chloride <30 mmol/L Through Week 24 43
Table S12. Post hoc Shift Tables for Proportion of Participants With Sweat Chloride <30 mmol/L Through Week 24 (Pooled From Trials VX20-121-102 and VX20-121-103) 44
Table S13. Other Secondary Efficacy Endpoints. 45
Table S14. Serious Adverse Events That Occurred in Two or More Participants in Either Group in Pooled Trials VX20-121-102 and VX20-121-103. 46

Table S15. Adverse Events That Led to Treatment Discontinuation in at Least 2 Participants in Either Group in Pooled Trials VX20-121-102 and VX20-121-103. 46

Trials VX20-121-102 and VX20-121-103 46

Table S16. Summary of Elevated Transaminases Events and Liver Function Test Enzyme Elevations in Pooled Trials VX20-121-102 and VX20-121-103.† 47

Table S17. Summary of Rash Events in Pooled Trials VX20-121-102 and VX20-121-103.* 48

Table S18. Summary of CK Elevation Events in Pooled Trials VX20-121-102 and VX20-121-103.... 48

Table S19. Summary of Neuropsychiatric Events in Pooled Trials VX20-121-102 and VX20-121-103.* 49

Table S20. Neuropsychiatric Adverse Events in Pooled Trials VX20-121-102 and VX20-121-103. ... 49

Table S21. Post hoc Summary: Depression-related Events in Pooled Trials VX20-121-102 and VX20-121-103.* 50

Table S22. Summary of Blood Pressure Measurements. 50

References 51

List of Site Investigators

The VX20-121-102 and VX20-121-103 Study Groups included Stefanie Krick, University of Alabama at Birmingham, Birmingham, Alabama; Karen McCoy, Nationwide Children’s Hospital, Columbus, Ohio; Christopher Goss, University of Washington Medical Center, Seattle, Washington; Daniel Weiner, UPMC Children's Hospital of Pittsburgh, Pittsburgh, Pennsylvania; Joanne Billings, University of Minnesota, Minneapolis, Minnesota; Clement Ren, Children's Hospital of Philadelphia, Philadelphia, Pennsylvania; Jeffrey Atkinson, Washington University School of Medicine / St. Louis Children's Hospital, St Louis, Missouri; Rachel Linnemann, Emory University, Atlanta, Georgia; Aaron Trimble, Oregon Health & Science University, Portland, Oregon; Christopher Fortner, SUNY Upstate Medical University, Syracuse, New York; Carla Frederick, CF Therapeutics Development Center of Western New York, Buffalo, New York; Theodore Liou, University of Utah, Salt Lake City, Utah; Hugo Escobar, The Children's Mercy Hospital, Kansas City, Missouri; Janice Wang, Long Island Jewish Medical Center, New Hyde Park, New York; Ashley Deschamps, Nebraska Medical Center, Omaha, Nebraska; Deborah Froh, University of Virginia Health System, Charlottesville, Virginia; James Chmiel, Riley Hospital for Children at Indiana University Health, Indianapolis, Indiana; Mark Wurth, University of Kentucky, Lexington, Kentucky; Lara Bilodeau, Institut Universitaire de Cardiologie et Pneumologie de Quebec - Universite Laval, Québec; Lael Yonker, Massachusetts General Hospital Cystic Fibrosis Center, Boston, Massachusetts; Allen Lapey, Massachusetts General Hospital Cystic Fibrosis Center, Boston, Massachusetts; Manu Jain, Northwestern Memorial Hospital, Chicago, Illinois; Susan Millard, Helen DeVos Children's Hospital CF Center, Grand Rapids, Michigan; James Tolle, Vanderbilt University Medical Center, Nashville, Tennessee; Kathryn Moffett, West Virginia University, Morgantown, West Virginia; Amy Filbrun, Michigan Medicine, Ann Arbor, Michigan; Shijing Jia, Michigan Medicine, Ann Arbor, Michigan; Jennifer Taylor-Cousar, National Jewish Health, Denver, Colorado; Fadel Ruiz, Texas Children's Hospital, Houston, Texas; Cynthia Brown, Indiana University, Indianapolis, Indiana; Bryon Quick, Kaiser Permanente, Oakland, California; Perry Brown, St. Luke's Cystic Fibrosis Center of Idaho, Boise, Idaho; John Kennedy, Boston Children's Hospital, Boston, Massachusetts; Ahmet Uluer, Boston Children's Hospital, Boston, Massachusetts; Jennifer Ruddy, ProMedica Toledo Hospital/Toledo Children's Hospital/Pediatric Pulmonary & Cystic Fibrosis Center, Toledo, Ohio; Edmund Sears, Maine Medical Partners, Portland, Maine; Boris Shkolnik, Albany Medical College, Albany, New York; Krishna Pancham, Cook Children's Health Care System, Fort Worth, Texas; Emily DiMango, Columbia University Medical Center, New York, New York; Claire Keating,

Columbia University Medical Center, New York, New York; Stanley Fiel, Morristown Medical Center, Morristown, New Jersey; Gary Mueller, Dayton Children's Hospital, Dayton, Ohio; Floyd Livingston, Nemours Children's Hospital, Orlando, Florida; Gregory Omlor, Akron Children's Hospital, Akron, Ohio; Andrew Braun, University of Wisconsin Hospital and Clinics, Madison, Wisconsin; Brian O'Sullivan, Dartmouth Hitchcock Medical Center, Lebanon, New Hampshire; Nighat Mehdi, University of Oklahoma Health Sciences Center, Oklahoma City, Oklahoma; Maria Gabriela Tupayachi Ortiz, University of Miami Miller School of Medicine, Miami, Florida; Patricia Dubin, Children's Foundation Research Center / Le Bonheur Children's Hospital, Memphis, Tennessee; Charlotte Teneback, Vermont Lung Center, Colchester, Vermont; Ted Kremer, UMass Memorial Medical Center, Worcester, Massachusetts; John Welter, New York Medical College, Valhalla, New York; Barbara Messore, Malattie Apparato Respiratorio 2 - Centro Fibrosi Cistica, Turin; Jordana Hoppe, Children's Hospital of Colorado, Aurora, Colorado; Raksha Jain, The University of Texas Southwestern Medical Center, Dallas, Texas; Allison Lambert, Providence Pediatric Pulmonary & Cystic Fibrosis Clinic, Spokane, Washington; Zubin Mukadam, Harper University Hospital, Detroit, Michigan; Francisco Calimano, Central Florida Pulmonary Group, PA, Orlando, Florida; Jimmy Johannes, Miller Children’s Hospital / Long Beach Memorial, Long Beach, California; Cori Daines, Banner University of Arizona Medical Center, Tucson, Arizona; Marie Egan, Yale New Haven Hospital, New Haven, Connecticut; Deanna Green, Johns Hopkins All Children's Hospital Outpatient Care Center, St. Petersburg, Florida; Thomas Keens, Children's Hospital Los Angeles, Los Angeles, California; Cesar Trillo-Alvarez, University of Florida, Shands Hospital, Gainesville, Florida; Andrew Wilhelm, University of Mississippi Medical Center, Jackson, Mississippi; Herschel Scher, Joe DiMaggio Cystic Fibrosis & Pulmonary Center, Hollywood, Florida; Joel Mermis, University of Kansas Medical Center, Kansas City, Kansas; Brian Morrissey, University of California Davis Medical Center, Sacramento, California; Santiago Reyes, Integris Health Baptist Medical Center, Oklahoma City, Oklahoma; David Schaeffer, Nemours Children's Specialty Care, Jacksonville, Florida; James Wallace, Sanford Children's Speciality Clinic, Sioux Falls, South Dakota; Christopher Barrios, Cardinal Glennon Children's Hospital - St. Louis University, St. Louis, Missouri; John Callison, University of Tennessee Medical Center, Knoxville, Tennessee; Zachary Holliday, Children’s Hospital University of Missouri Health Sciences Center, Columbia, Missouri; Ross Klingsberg, Tulane Medical Center, New Orleans, Louisiana; Zsolt Szepfalusi, Medizinische Universität Wien, Vienna; Ralph Epaud, Centre Hospitalier Intercommunal Creteil, Creteil; Sylvie Leroy, Centre Hospitalier Universitaire (CHU) de Nice - Hopital Pasteur, Nice; Reta Fischer Biner, Lindenhofspital

- Quartier Bleu, Bern; Alexander Moeller, Kinderspital Zuerich, Zürich; Donatello Salvatore, Centro Regionale Fibrosi Cistica, A.O. Ospedale San Carlo, Potenza; Ori Efrati, Sheba Medical Center - The Edmond and Lili Safra Children's Hospital, Tel Aviv; Malana Cohen, Hadassah University Hospital Mount Scopus, Jerusalem; Dario Prais, Schneider Children's Medical Center of Israel, Central; Pilar Azevedo, Hospital de Santa Maria, Lisbon; Esther Quintana Gallego, Hospital Universitario Virgen del Rocio, Sevilla; Larry Johnson, University of Arkansas for Medical Sciences, Little Rock, Arkansas; Janice Wong, Waikato Hospital, Hamilton; Sophie Gohy, Cliniques Universitaires Saint-Luc, Brussels; Andrea Gramegna, Fondazione IRCCS Ospedale Maggiore Policlinico, Mangiagalli e Regina Elena, Milan; Pedro Mondejar-Lopez, Hospital Virgen de la Arrixaca, Murcia; Alejandro Lopez Neyra, Hospital Infantil Universitario Nino Jesus, Madrid; Hanne Olesen, Aarhus University Hospital, Aarhus; Adrien Tissot, Centre Hospitalier Universitaire De Nantes - G. R. Laennec, Nantes; Christophe Marguet, CHU de Rouen - Hopital Charles Nicolle, Rouen; Adelina Amorim, Hospital Sao Joao, Porto; Sheila Sivam, Royal Prince Alfred Hospital, Camperdown; Oscar Asensio de la Cruz, Corporacio Sanitaria Parc Tauli - Sabadell Hospital Universitari, Barcelona; Maya Desai, Birmingham Children's Hospital, Birmingham; Michael Waller, King's College Hospital, London; Louisa Owens, Sydney Children's Hospital, Sydney; Caralee Forseen, Augusta University, Augusta, Georgia; Tamizan Kherani, Stollery Children's Hospital, Edmonton; Chad Marion, Wake Forest Baptist Health, Winston-Salem, North Carolina; Anna-Maria Dittrich, Medizinische Hochschule Hannover, Hannover; Jerimiah Lysinger, Billings Clinic, Billings, Montana; Dorota Sands, Klinika Mukowiscydozy IMD Oddozial Chorob Pluc Szpzoz IM. Dzieci WarszaWY, Lomianki; Egil Bakkeheim, Oslo University Hospital, Department of Paediatric Medicine, Oslo; Elizabeth Tullis, St. Michael's Hospital, Toronto; Mark Chilvers, British Columbia Children's Hospital, Vancouver; Bradley Quon, St. Paul's Hospital, Vancouver; Larry Lands, McGill University Health Centre, Glen Site, Montreal Children's Hospital, Montreal; Michael Parkins, University of Calgary Medical Clinic of the Foothills Medical Centre, Calgary; Francois Tremblay, Centre Hospitalier de l'Universite de Montreal (CHUM) Hotel-Dieu, Montreal; Anirban Maitra, Royal Manchester Children's Hospital, Manchester; Brian Casserly, University Hospital Limerick (Adults), Limerick; Heather Staples, Prisma Health Richland Campus, Columbia, South Carolina; Okan Elidemir, Nemours Children's Specialty Care, Pensacola, Pensacola, Florida; Carmen Luna, Hospital Universitario 12 de Octubre, Madrid; Elpis Chatziagorou, General Hospital of Thessaloniki 'Hippokratio', 3rd Pediatrics Department of Aristotle University of Thessaloniki, Thessaloniki; Pornchai Tirakitsoontorn, Children's Hospital of Orange County, Orange, California; Dominique

Grenet, Hopital Foch (Suresnes), Hopital Foch, Adultes, Suresnes; Sarah Brown, St. Bartholomew’s Hospital, London; Barry Linnane, University Hospital Limerick (Pediatrics), Limerick; Jonathan Singer, University of California San Francisco, Lung Transplant Program, San Francisco, California; Dennis Schellhase, Atrium Health Levine Children's Hospital, Charlotte, North Carolina; Jon Roberts, Driscoll Children's Hospital, Corpus Christi, Texas; John Michael Nicholson, St. Joseph's Health Care London, London; Katerina Manika, General Hospital of Thessaloniki “Papanikolaou”, Thessaloniki; Christopher King, Inova Fairfax, Falls Church, Virginia; Aurelie Tatopoulos, Centre hospitalier universitaire de Nancy, Hopital de Braboi, Vandœuvre-lès-Nancy ; Catherine Byrnes, Starship Children's Hospital, Auckland; Felix Ringshausen, Medizinische Hochschule Hannover, Hannover; Helge Hebestreit, Universitatsklinikum Wurzburg, Würzburg; Michael Lorenz, Mukoviszidose-Zentrum am Universitatsklinikum Jena, Klinik fur Kinder- und Jugendmedizin, Jena; Renate Ruppel, Friedrich-Alexander University of Erlangen-Nuremberg, University Children's Hospital, Erlangen; Pavel Drevinek, Fakultni nemocnice v Motole, Prague; Stefanie Dillenhofer, St. Josef-Hospital, Bochum; Mirjam Stahl, Charite Paediatric Pulmonology Department, Berlin; Wolfgang Gleiber, Johann Wolfgang Goethe University, Frankfurt; Lukas Homola, Klinika Detskych Infekcnich Nemoci, Brno; Tacjana Pressler, Juliane Marie Center, Rigshospitalet, Copenhagen; Susanne Naehrig, Klinikum Innenstadt, University of Munich, Munich; Florian Stehling, Kinderklinik III, Abt. fur Pneumologie, Essen; Sivagurunathan Sutharsan, Ruhrlandklinik Westdeutsches Lungenzentrum am Klinikum Essen, Essen; Azadeh Bagheri-Potthoff, Justus-Liebig-Universität Gießen Zentrum für Kinderheilkunde und Jugendmedizin, Gießen; Rainald Fischer, Pneumologisches Studienzentrum Muenchen-West, Munich; Krystyna Poplawska, Johannes Gutenberg-Universitaet, Mainz; Charles Haworth, Royal Papworth Hospital NHS Foundation Trust, Cambridge; Nicholas Simmonds, Royal Brompton Hospital, London; Don Urquhart, Royal Hospital for Sick Children, Edinburgh; James Duckers, All Wales Adult Cystic Fibrosis Centre, University Hospital Llandough, Cardiff; Julian Legg, Southampton General Hospital, Southampton; Mary Carroll, Southampton General Hospital, Southampton; Peter Barry, Wythenshawe Hospital, Manchester; Alexander Horsley, Wythenshawe Hospital, Manchester; Simon Doe, Clinical Research Facility, Newcastle upon Tyne; Tom Hilliard, University Hospitals Bristol and Weston NHS Foundation Trust, Bristol Royal Hospital, Bristol; Gordon MacGregor, Clinical Research Facility, Queen Elizabeth University Hospital, Glasgow; Neil Patel, Birmingham Heartlands Hospital, Birmingham; Nicholas Withers, Royal Devon University Healthcare NHS Foundation Trust, Exeter; Daniel Peckham, St. James University Hospital, Leeds; Martin Ledson, Liverpool Heart and Chest Hospital, Liverpool; Timothy Lee, Leeds

General Infirmary, Leeds; Usma Koser, Western General Hospital, Edinburgh; Veronique Houdouin, Hopital Robert Debre, Paris; Sophie Ramel, Centre de Perharidy, Roscoff; Isabelle Fajac, Hopital Cochin, Paris; Isabelle Sermet-Gaudelus, Hopital Necker, Enfants Malades, Paris; Lieke Kamphuis, Erasmus Medical Center, Rotterdam; Kors van der Ent, University Medical Center, Utrecht, Department of Pulmonology and Tuberculosis, Utrecht; Francois Vermeulen, Universitaire Ziekenhuizen Leuven - Campus Gasthuisberg, Leuven; Raphael Chiron, Hopital Arnaud de Villeneuve, Montpelier; Philippe Reix, CHU Lyon - Hopital Femme Mere-Enfant, Lyon; Eva Van Braeckel, Universitair Ziekenhuis Gent, Brussels; Christiane Knoop, Cliniques Universitaires de Bruxelles Hopital Erasme, Brussels; Eef Vanderhelst, Universitair Ziekenhuis Brussel - Campus Jette, Brussels; Isabelle Durieu, Centre Hospitalier Lyon Sud, Lyon; Stephanie Bui, Groupe Hospitaler Pellegrin, CHU De Bordeaux, Bordeaux; Laurence Weiss, Hôpital de Hautepierre, AX5; Strasbourgt; Olivier Le Rouzic, Institut Cœur Poumon, CHU de Lille, Lille; Renske van der Meer, HagaZiekenhuis van den Haag, Den Haag; Jolt Roukema, UMC St. Radboud, Nijmegen; Josje Altenburg, Academisch Medisch Centrum (Academic Medical Centre), Amsterdam; Silvia Gartner, Hospital Universitari Vall d Hebron, Barcelona; Enrique Blitz Castro, Hospital Universitario Ramon y Cajal, Madrid; Amparo Sole Jover, Hospital Universitario y Politecnico La Fe, Valencia; Desmond Cox, Children's Health Ireland at Crumlin, Dublin; Edward McKone, St. Vincent's University Hospital, Dublin; Barry Plant, Cork University Hospital, Cork; Michael Williamson, Children's Health Ireland at Temple Street, Dublin; Basil Elnazir, Children's Health Ireland at Tallaght, Dublin; Telma Barbosa, CHP - Hospital de Santo Antonio, Porto; Maria Cols Roig, Hospital Saint Joan de Deu, Barcelona; Philip Robinson, The Royal Children's Hospital, Melbourne; Lucy Burr, Mater Adult Hospital, Brisbane; Claire Wainwright, Mater Adult Hospital, Brisbane; Daniel Henderson, The Prince Charles Hospital, Brisbane; Siobhain Mulrennan, Institute for Respiratory Health, Perth; Barry Clements, Telethon Kids Institute, Perth; Peter Middleton, Westmead Hospital, Sydney; Tom Kotsimbos, Alfred Hospital, Melbourne; Giovanni Taccetti, Azienda Ospedaliero Universitaria Ospedale Pediatrico Meyer, Florence; Marco Cipolli, Azienda Ospedaliera di Verona - Ospedale Civile Maggiore, Verona; Valeria Dacco, Fondazione IRCCS Ca’ Granda Ospedale Maggiore Policlinico, Milan; Benedetta Fabrizzi, Azienda Ospedaliero Universitaria Ospedale Riuniti, Ancona; Carlo Castellani, IRCCS Istituto Giannina Gaslini-Ospedale Pediatrico, Genova; Ernst Eber, University of Graz, Graz; Dorothea Appelt, Medizinische Universität Innsbruck, Innsbruck; Michael Studnicka, Uniklinikum Salzburg - Universitätsklinik für Pneumologie/Lungenheilkunde, Salzburg; Isabelle De Monestrol, Karolinska Universitetssjukhuset, Huddinge, Stockholm; Marita Gilljam, Sahlgrenska Universitetssjukhuset, Gothenburg; Ewa

Sapiejka, Pediatric Hospital Polanki named of Maciej Płażyński, Gdansk; Henryk Mazurek, Institute of Tuberculosis and Lung Diseases, Rabka-Zdroj ; Ulrika Lindberg, Lund University Skanes Universitetssjukhus, Lund; Mette Engan, Haukeland Universitetssjukehus (CF), Bergen; Carsten Schwarz, Klinikum Westbrandenburg (CF), Potsdam; Alain Sauty, Réseau Hospitalier Neuchâtelois, Neuchâtel; Sebastian Bode, Universitatsklinikum Ulm, Klinik fur Kinder- und Jugendmedizin (CF), Ulm; Julie Mankikian, Hopital Bretonneau, Tours; Michael Epton, Canterbury District Health Board, Christchurch; Adrien Halasz, National Koranyi Institute for TBC and Pulmonology, Budapest; Istvan Laki, Pulmonology Institute Torokbalint, Torokbalint; Mark O'Carroll, Auckland City Hospital, Auckland.

Supplementary Methods

In Vitro Assay of CFTR Function

The Fischer rat thyroid (FRT) system is a clinically validated in vitro assay which measures CFTR-mediated chloride transport to assess responsiveness of *CFTR* variants to CFTR modulators. The FRT system is a stable expression system integrating mutated *CFTR* cDNA as a single copy into the same genomic location using the Invitrogen Flp-In™ system. Ussing chamber studies of cells stably expressing a *CFTR* variant were used to measure responsiveness of 128 variants to vanzacaftor–tezacaftor–deutivacaftor.

The effect of vanzacaftor–tezacaftor–deutivacaftor or elexacaftor-tezacaftor-ivacaftor on chloride transport for different *CFTR* variants was evaluated in vitro as previously described.[1-4] Briefly, CFTR was stimulated by addition of forskolin (10 µM) and then subsequently inhibited using a cocktail of CFTR inhibitors. The short circuit current (e.g., current attributable to CFTR) was calculated as the peak forskolin-stimulated response minus the minimum stable chloride current after addition of the CFTR inhibitor cocktail. The minimal detectable chloride current in Flp-In™-FRT without an introduced *CFTR* gene is 4 ± 1 $\mu A/cm^2$, and this value was considered equivalent to that of cells lacking CFTR-mediated chloride transport. The current induced by wild type CFTR was also measured. For each variant, chloride transport is determined as $\mu A/cm^2$ and as a percent of wild type CFTR (% normal). Percent normal was calculated by dividing the $\mu A/cm^2$ value of the *CFTR* variant form by the $\mu A/cm^2$ value of baseline normal CFTR. Values for each *CFTR* variant were determined from six valid, independent experiments.

Trials VX20-121-102 and VX20-121-103 Eligibility Criteria

The trials were conducted in accordance with the Declaration of Helsinki, local applicable laws and regulations, and current Good Clinical Practice Guidelines as described by the International Council for Harmonization.

Inclusion Criteria

1. Participants (or legally appointed and authorized representative) signed and dated an informed consent form, and when appropriate, an assent form.
2. Willing and able to comply with scheduled visits, treatment plan, study restrictions, laboratory tests, contraceptive guidelines, and other trial procedures.
3. Participants aged 12 years or older, on the date of informed consent.
4. Confirmed diagnosis of cystic fibrosis as determined by the investigator.
5. **Trial VX20-121-102 Only:** Heterozygous for *F508del* and a minimal function mutation, defined as a mutation that either results in no translated CFTR protein or that is non-responsive to tezacaftor, ivacaftor, or tezacaftor/ivacaftor based on in vitro testing (*F508del*-minimal function genotypes) (**Table S1**). If the screening *CFTR* genotype result was not received before randomization, a previous *CFTR* genotype laboratory report may have been used to establish eligibility. Participants who were enrolled and whose screening genotype did not confirm study eligibility were discontinued from the trial.
6. **Trial VX20-121-103 Only:** Participant had one of the following genotypes: 1) homozygous for *F508del*; 2) heterozygous for *F508del* and a gating mutation; 3) heterozygous for *F508del* and a residual function mutation; 4) elexacaftor-tezacaftor-ivacaftor-responsive-non-*F508del*. If the screening *CFTR* genotype result was not received before randomization,

a previous *CFTR* genotype laboratory report may have been used to establish eligibility. Participants who were enrolled and whose screening genotype did not confirm study eligibility were discontinued from the trial.

7. **Trial VX20-121-102 Only:** For participants who were receiving elexacaftor-tezacaftor-ivacaftor, FEV_1 value ≥40% and ≤90% of predicted mean for age, sex, and height (equations of the Global Lung Function Initiative [GLI]) at the Screening Visit.[5] All participants who were not receiving elexacaftor-tezacaftor-ivacaftor had an FEV_1 value ≥40% and ≤80% of predicted mean. Spirometry measurements met American Thoracic Society/European Respiratory Society criteria for acceptability and repeatability.[6]

8. **Trial VX20-121-103 Only:** For participants who were receiving Vertex CFTR modulator therapy, FEV_1 value ≥40% and ≤90% of predicted mean for age, sex, and height (equations of the GLI) at the Screening Visit.[5] All participants who were not receiving Vertex CFTR modulator therapy had an FEV_1 value ≥40% and ≤80% of predicted mean. Spirometry measurements met American Thoracic Society/European Respiratory Society criteria for acceptability and repeatability.[6]

9. Stable CF disease as judged by the investigator.

10. Willing to remain on a stable CF treatment regimen through completion of trial participation.

Exclusion Criteria

1. History of any comorbidity that, in the opinion of the investigator, may have confounded the results of the study or posed an additional risk in administering study drug(s) to the participant. This included, but was not limited to, the following:

- Hepatic cirrhosis with portal hypertension, moderate hepatic impairment (Child Pugh Score 7 to 9), or severe hepatic impairment (Child Pugh Score 10 to 15).
- Solid organ or hematological transplantation.
- Alcohol or drug abuse in the past year, including, but not limited to, cannabis, cocaine, and opiates, as deemed by the investigator.
- Cancer, except for squamous cell skin cancer, basal cell skin cancer, and Stage 0 cervical carcinoma in situ (all 3 with no recurrence for the last 5 years).

2. History of intolerance to study drug that would have posed an additional risk to the participant in the opinion of the investigator (e.g., participants with a history of liver function test [LFT] elevations requiring treatment interruption or discontinuation, allergy or hypersensitivity to the study drug).

3. Any of the following abnormal laboratory values at screening:

- Hemoglobin less than 10 g/dL
- Total bilirubin greater than or equal to 2 times the upper limit of normal
- Aspartate aminotransferase (AST), alanine aminotransferase (ALT), γ-glutamyl transferase (GGT), or alkaline phosphatase (ALP) greater than or equal to 3 times the upper limit of normal
- Abnormal renal function defined as glomerular filtration rate ≤50 mL/min/1·73 m^2
- (calculated by the Modification of Diet in Renal Disease Study Equation) for participants ≥18 years of age and ≤45 mL/min/1·73 m^2 (calculated by the Counahan-Barratt equation) for participants aged 12 to 17 years (inclusive).[7-9]

4. An acute upper or lower respiratory infection, pulmonary exacerbation, or changes in therapy (including antibiotics) for sinopulmonary disease within 28 days before the first dose of elexacaftor-tezacaftor-ivacaftor in the run-in period (day -28).

5. Lung infection with organisms associated with a more rapid decline in pulmonary status (including, but not limited to, *Burkholderia cenocepacia*, *Burkholderia dolosa*, and *Mycobacterium abscessus*). For participants who had a history of a positive culture, the investigator applied the following criteria to establish whether the participant was free of infection with such organisms:

   - The participant had not had a respiratory tract culture positive for these organisms within the 12 months before the date of informed consent.
   - The participant had at least 2 respiratory tract cultures negative for such organisms within the 12 months before the date of informed consent, with the first and last of these separated by at least 3 months, and the most recent one within the 6 months before the date of informed consent.

6. An acute illness not related to cystic fibrosis (e.g., gastroenteritis) within 14 days before the first dose of elexacaftor-tezacaftor-ivacaftor in the run-in period (day -28).

7. Ongoing or prior participation in a study of an investigational treatment other than a Vertex CFTR modulator within 28 days or 5 terminal half-lives (whichever is longer) before screening, or participation in an interventional study of a non-investigational treatment from screening through end of study participation. The duration of the elapsed time may have been longer if required by local regulations.

8. Use of prohibited medications within the specified window before the first dose of elexacaftor-tezacaftor-ivacaftor in the run-in period (day -28).

9. Pregnant or breast-feeding females. Female participants had a negative pregnancy test at screening (serum test) and run-in period/day -28 (urine test).

10. The participant or a close relative of the participant was the investigator or a subinvestigator, research assistant, pharmacist, study coordinator, or other staff directly involved with the conduct of the study at that site. However, an adult (aged 18 years or older) who is a relative of a study staff member may have enrolled in the study provided that

    - the adult lived independently of and did not reside with the study staff member, and
    - the adult participated in the trial at a site other than the site at which the family member was employed.

Follow-up

A safety follow-up visit was scheduled to occur approximately 28 (± 7) days after the last dose of study drug for participants who completed study drug dosing and for participants who prematurely discontinued study drug dosing. The safety follow-up visit was not required for participants who completed the Treatment Period and transitioned within 28 days of the last dose of study drug to either:

- a commercially available Vertex CFTR modulator regimen,
- a managed access program-supplied Vertex CFTR modulator regimen, or
- an open-label study or other qualified Vertex study.

If a participant prematurely discontinued study drug treatment, an early termination of treatment visit was to be scheduled as soon as possible after the decision to discontinue treatment. Participants who prematurely discontinued treatment were also required to complete the safety follow-up visit. Participants who prematurely discontinued treatment were not eligible to enroll in the open-label extension safety study. If a participant withdrew consent for the study, no further assessments were performed.

Treatment Interruption and Stopping Rules

Modifications of the study drug dose were prohibited. Treatment may have been interrupted as outlined below. If any unacceptable toxicity arose, individual participants were to discontinue dosing.

Liver function tests

The central laboratory notified the medical monitor of ALT or AST >3 × ULN and total bilirubin >2 × ULN that were derived from centrally submitted samples.

Participants with new treatment-emergent ALT or AST elevations of >3 × ULN, with or without total bilirubin >2 × ULN, were followed closely, including confirmatory testing performed by the central laboratory within 48 to 72 hours of the initial finding and subsequent close monitoring of ALT, AST, and bilirubin levels, as clinically indicated.

Study drug administration was interrupted immediately (prior to confirmatory testing) if any of the following criteria were met:

- ALT or AST >8 × ULN
- ALT or AST >5 × ULN for more than 2 weeks

- ALT or AST >3 × ULN, in association with total bilirubin >2 × ULN and/or clinical jaundice

A thorough investigation of potential causes was to be conducted, and the participant was followed closely for clinical progression.

Study drug administration was discontinued if the following criterion was met:

- Subsequent ALT or AST values confirmed the initial elevation that satisfied the interruption rule (above), and no convincing alternative etiology (e.g., acetaminophen use, viral hepatitis, alcohol ingestion) was identified, regardless of whether transaminase levels had improved

All participants in whom treatment was discontinued for elevated transaminases (and bilirubin, as applicable) were to have these levels monitored closely until levels normalized or returned to baseline.

If an alternative, reversible cause of transaminase elevation with or without increased bilirubin or clinical jaundice was identified, study drug administration may have been resumed once transaminases returned to baseline or were ≤2 × ULN, whichever was higher. Upon resumption of study drug, transaminases and bilirubin were to be assessed weekly for 4 weeks. If a protocol-defined transaminase elevation interruption threshold recurred within 4 weeks of rechallenge with the study drug (with confirmation of the initial elevation by repeat testing within 48 to 72 hours), then the study drug was permanently discontinued, regardless of the presumed etiology.

Rash

Participants who developed a generalized rash were monitored closely. Study drug dosing was to be interrupted if a participant developed a generalized rash of Grade 3 or higher, or a rash that

was considered a serious adverse event. The participant may have resumed study drug treatment if considered clinically appropriate by the investigator.

Schedule of Assessments (Trials VX20-121-102 and VX20-121-103)

Event/Assessment[1]	Run-in Period		Treatment Period						ETT Visit	Safety Follow-up Visit 28 (± 7) Days After the Last Dose of Study Drug
	Day -28 (± 1 Day)	Day -14 (Day -15 to Day -3)	Day 1	Day 15 (± 3 Days)	Weeks 4, 8, 12, 16 (± 5 Days)	Weeks 20, 28, 32, 40, 44, 48 (± 5 Days)	Weeks 24 and 36 (± 5 Days)	Week 52 (± 5 Days)		
CFQ-R			X		Weeks 8 and 16		Week 24	X	X	
Height and weight	X		X	X	Weeks 4, 8, 16		X	X	X	X
Ophthalmologic examination								X	X	
Physical examination	Abbrev		Complete					Complete	Complete	
Pregnancy testing	Urine		Urine		Urine	Urine	Urine	Urine	Serum	Serum
Standard 12-lead ECG	X		X	X	Weeks 4, 8, 16		X	X	X	X
Vital signs	X		X	X	X		X	X	X	X
Pulse oximetry	X		X	X	X		X	X	X	X
Spirometry		X	X	X	Weeks 4, 8, 16		X	X	X	X
Sweat chloride		X	X	X	Weeks 4 and 16		X	X	X	
Serum chemistry	X		X[2]	X	X		X	X	X	X
Hematology	X		X[2]	X	X		X	X	X	X
Coagulation	X		X[2]		Week 12		X	X	X	X
Urinalysis	X		X		Week 12		X	X	X	X
Run-in ELX/TEZ/IVA dosing	Day -28 through Day -1									
Randomized study drug dosing			Day 1 through evening before Week 52 Visit							
Other events related to outcome	Continuous from signing of ICF through completion of study participation									
Medications review	Continuous from signing of ICF through completion of study participation									
Treatments and procedures review	Continuous from signing of ICF through completion of study participation									
AEs and SAEs	Continuous from signing of ICF through completion of study participation									

AE: adverse event; CFQ-R: CF Questionnaire-Revised; ELX: elexacaftor; ETT: Early Termination of Treatment; IVA: ivacaftor; PE: physical examination; SAE: serious adverse event; TEZ: tezacaftor;

[1] All assessments were performed before dosing unless noted otherwise.
[2] Blood samples were collected before the first dose of study drug in the Treatment Period.

Statistical Analysis

Primary Endpoint

The primary estimand was defined as the following:

- Treatment: vanzacaftor–tezacaftor–deutivacaftor versus elexacaftor-tezacaftor-ivacaftor
- Population: Study population represented by inclusion and exclusion criteria
- Variable: Absolute change from baseline (after elexacaftor-tezacaftor-ivacaftorrun-in) in FEV_1 % predicted through Week 24 (estimated by averaging weeks 16 and 24)
- Handling of intercurrent events:

The treatment policy strategy was used to handle the use of non-study drug CFTR modulators for >3 days in either the run-in period or the treatment period prior to week 24, which means that observed FEV_1 % predicted values were used, if available, after this prohibited medication use.

The treatment policy strategy was used to handle treatment discontinuation prior to Week 24, which means that observed FEV_1 % predicted values were used, if available, after treatment discontinuation.

- Population level summary: Difference in variable means between vanzacaftor–tezacaftor–deutivacaftor and elexacaftor-tezacaftor-ivacaftor groups

Key Secondary Endpoints

The estimand for the absolute change from baseline in sweat chloride through Week 24 was defined as the following:

- Treatment: vanzacaftor–tezacaftor–deutivacaftor versus elexacaftor-tezacaftor-ivacaftor

- Population: Trial population represented by inclusion and exclusion criteria
- Variable: Absolute change from baseline in sweat chloride through week 24 (estimated by averaging weeks 16 and 24)
- Handling of intercurrent events: Same as the primary estimand
- Population level summary: Difference in variable means between vanzacaftor–tezacaftor–deutivacaftor and elexacaftor-tezacaftor-ivacaftor groups

The estimand for the proportion of subjects with sweat chloride <60 mmol/L through Week 24 was defined as the following:

- Treatment: vanzacaftor–tezacaftor–deutivacaftor versus elexacaftor-tezacaftor-ivacaftor
- Population: Pooled study population represented by inclusion and exclusion criteria
- Variable: Response defined as sweat chloride <60 mmol/L through Week 24 (estimated by averaging weeks 16 and 24)
- Handling of intercurrent events: Same as the primary estimand
- Population level summary: Odds ratio comparing the response rates in vanzacaftor–tezacaftor–deutivacaftor and elexacaftor-tezacaftor-ivacaftor groups

The estimand for the pooled analysis of proportion of subjects with sweat chloride <30 mmol/L through Week 24 was defined similarly to that for <60 mmol/L.

Testing Hierarchy

The testing order of the key secondary endpoints within each trial was as follows:

- Absolute change from baseline in sweat chloride concentration through week 24 (full analysis set)
- Proportion of participants with sweat chloride concentration <60 mmol/L through week 24 (pooled full analysis set)
- Proportion of participants with sweat chloride concentration <30 mmol/L through week 24 (pooled full analysis set)

Other Secondary Endpoints

The secondary endpoint of absolute change from baseline in CFQ-R RD score through week 24 was analyzed using an MMRM model similar to the primary endpoint.

The secondary endpoint of number of pulmonary exacerbation (defined below) through week 52 was analyzed descriptively and the pulmonary exacerbation rate per year was presented, along with difference in pulmonary exacerbation rate between treatment groups and the associated 95% CI.

Other secondary endpoints which were continuous were analyzed using an MMRM similar to the analysis of the primary efficacy endpoint. The endpoints based on proportion of participants with sweat chloride response were analyzed using a GEE model similar to the analysis of key secondary endpoint.

Protocol-defined pulmonary exacerbation were any new or change in antibiotic therapy (intravenous, inhaled, or oral) for any 4 or more of the following signs/symptoms. This definition

is based on the definition of a pulmonary exacerbation used in previous clinical studies, including ivacaftor clinical trials.[11, 12]

- Change in sputum
- New or increased hemoptysis
- Increased cough
- Increased dyspnea
- Malaise, fatigue, or lethargy
- Temperature above 38°C (equivalent to approximately 100·4°F)
- Anorexia or weight loss
- Sinus pain or tenderness
- Change in sinus discharge
- Change in physical examination (PE) of the chest
- Decrease in pulmonary function by 10%
- Radiographic changes indicative of pulmonary infection

Post-hoc analysis

Post-hoc subgroup analyses of the primary endpoint by genotype in Trial VX20-121-103 (*F508del-F508del*, *F508del*-gating, *F508del*-residual function, and elexacaftor-tezacaftor-ivacaftor-responsive-non-*F508del* genotypes) was implemented using an MMRM similar to the primary analysis. For the first key secondary endpoint (absolute change from baseline in sweat chloride through Week 24), subgroup analyses for the same subgroups as the primary endpoint

were implemented as part of post-hoc analysis. Additional post-hoc analysis for this endpoint included subgroup analyses within Trials VX20-121-102 and VX20-121-103 in participants who had sweat chloride <30 mmol through week 24 (regardless of their baseline sweat chloride value) and in participants who had sweat chloride ≥30 mmol/L at baseline and then achieved sweat chloride <30 mmol/L through week 24. To assess the robustness of the findings from the key secondary endpoints of proportion of participants with sweat chloride <60 mmol/L or <30 mmol/L through week 24, shift tables were generated as post-hoc analyses to evaluate the percentage of participants with sweat chloride either above or below 60 mmol/L or 30 mmol/L at baseline compared to sweat chloride values through week 24. The count and percentage for each combination of baseline and post-baseline category were presented.

For the continuous variables in the baseline summary, the 1st and 3rd quartile (Q1 and Q3) were calculated to provide information on the interquartile range which is the difference between Q3 and Q1. Additionally, the duration of commercial elexacaftor-tezacaftor-ivacaftor use was summarized by pooling data from Trials VX20-121-102 and VX20-121-103. Post-hoc safety analyses for pooled safety data from Trials VX20-121-102 and VX20-121-103 using a customized medical query for depression-related events was conducted; count and percentage of participants were presented.

The distribution of time to first ALT or AST elevation of more than 3 times ULN for the pooled safety set was presented as a bar plot with each bar representing a 3-month interval and separate bars for the two treatment groups. The height of the bar represented the number of participants with first ALT or AST elevation of more than 3 times ULN in the corresponding quarter. A similar plot was presented for time to first rash event.

To assess the incidence of elevated transaminases and rash events in a CFTR modulator-naive population who received elexacaftor-tezacaftor-ivacaftor for 52 weeks, the data from the placebo-controlled, pivotal Phase 3 trial VX17-445-102 for elexacaftor-tezacaftor-ivacaftor was combined with its open-label extension trial VX17-445-105. All participants who received elexacaftor-tezacaftor-ivacaftor either in the parent study (VX17-445-102) or its extension study (VX17-445-105) were included in this analysis.

Adverse Event Definition and Reporting

All participants or participants' parents or legal guardians were queried, using nonleading questions, about the occurrence of adverse events at each study visit. When possible, a constellation of signs and/or symptoms were identified as 1 overall event or diagnosis.

An adverse event was defined as any untoward medical occurrence in a participant during the study; the event did not necessarily have a causal relationship with the treatment. This included any newly occurring event or worsening of a pre-existing condition (e.g., increase in its severity or frequency) after the ICF was signed.

The investigator determined and recorded the severity (mild, moderate, severe, life-threatening, death) of all serious and nonserious adverse events. The guidance available at the following website was consulted: Common Terminology Criteria for Adverse Events (CTCAE), Version 5.0, Cancer Therapy Evaluation Program, http://ctep.cancer.gov/protocolDevelopment/electronic_applications/ctc.htm (Accessed July 2021). When considering the severity of an adverse event in a pediatric subject, the investigator was to consider that reference ranges for pediatric clinical laboratory parameters may differ from those in the CTCAE.

The investigator also assessed the relationship of the adverse event, if any, to the study drug(s) (related, possibly related, unlikely related, and not related).

Protocol Deviations

An important protocol deviation (IPD) was defined as any protocol deviation that may have significantly impacted the completeness, accuracy, and/or reliability of key study data or that may have significantly affected a subject's rights, safety, or well-being.

In Trial VX20-121-102, a total of 20 (5·0%) participants had 21 IPDs described below.

- Eight participants had an IPD related to investigational product.
- Seven participants had an IPD related to prohibited concomitant medications.
- Three participants had an IPD related to eligibility criteria.
- Two participants had an IPD related to study conduct/procedures.
- One participant in the vanzacaftor–tezacaftor–deutivacaftor group had an IPD related to a delay in serious adverse event reporting.

In Trial VX20-121-103, a total of 27 (4·7%) participants had IPDs.

- Sixteen participants had an IPD related to investigational product.
- Five participants had an IPD related to eligibility criteria.
- Three participants had an IPD related to a delay in serious adverse event reporting.
- Two participants had an IPD related to study conduct/procedures.
- One participant in the elexacaftor-tezacaftor-ivacaftor group had an IPD related to informed consent.

- One participant in the elexacaftor-tezacaftor-ivacaftor group had an IPD related to prohibited concomitant medications.

Supplementary Results

In vitro FRT data

A total of 257 *CFTR* variants were evaluated in the FRT system. Prior testing demonstrated that 129 *CFTR* variants were responsive to at least 1 component of the vanzacaftor–tezacaftor–deutivacaftor combination. As responsiveness to a component means responsiveness to the combination, these 129 *CFTR* variants are therefore predicted to be responsive to vanzacaftor–tezacaftor–deutivacaftor. The other 128 *CFTR* variants were evaluated with both elexacaftor–tezacaftor–ivacaftor and vanzacaftor–tezacaftor–deutivacaftor; 97 of these were previously shown to be responsive to elexacaftor-tezacaftor-ivacaftor in vitro (and are approved for commercial use/received label indication in some regions), and 31 variants were not responsive to elexacaftor–tezacaftor–ivacaftorin vitro and are not approved for any CFTR modulator therapy. Of the 128 *CFTR* variants tested with vanzacaftor–tezacaftor–deutivacaftor, in vitro FRT analyses of chloride transport confirmed all 97 elexacaftor–tezacaftor–ivacaftor-responsive variants tested were also responsive to vanzacaftor–tezacaftor–deutivacaftor, with an additional 31 *CFTR* variants identified as responsive to vanzacaftor–tezacaftor–deutivacaftor and not elexacaftor–tezacaftor–ivacaftor (Table S8).

Post-hoc subgroup analysis for Trials VX20-121-102 and VX20-121-103 demonstrated that for participants with sweat chloride below 30 mmol/L through Week 24, absolute change from baseline through week 24 was greater in the vanzacaftor-tezacaftor-deutivacaftor group compared to the elexacaftor-tezacaftor-ivacaftor group. Similar results were observed when analysing participants with baseline sweat chloride greater than or equal to 30 mmol/L

Post-hoc subgroup analyses were conducted to evaluate the percentage of participants with sweat chloride concentration either above or below the thresholds of 30 mmol/L and 60 mmol/L at baseline compared to sweat chloride concentration through week 24 (appendix pp 46-47). Results demonstrate that vanzacaftor-tezacaftor-deutivacaftor was more effective compared to elexacaftor-tezacaftor-ivacaftor and led to a greater proportion of participants who had baseline sweat chloride concentrations greater than or equal to 60 mmol/L achieving sweat chloride

concentration below 60 mmol/L through week 24. Similarly, a greater proportion of participants with baseline sweat chloride concentration greater than or equal to 30 mmol/L at baseline achieved sweat chloride concentration below 30 mmol/L through week 24 following treatment with vanzacaftor-tezacaftor-deutivacaftor compared to elexacaftor-tezacaftor-ivacaftor. These post-hoc analyses also demonstrated that vanzacaftor-tezacaftor-deutivacaftor was more effective compared to elexacaftor-tezacaftor-ivacaftor at maintaining sweat chloride concentration for participants who had baseline sweat chloride concentrations either below 30 mmol/L or below 60 mmol/L.

Supplementary Figures

Figure S1. Subgroup Analysis of Absolute Change in $ppFEV_1$ From Baseline Through Week 24.

Pre-specified forest plot subgroup analysis of absolute change in $ppFEV_1$ from baseline through week 24 are presented below for Trial VX20-121-102 and VX20-Trial 121-103.

Trial VX20-121-102

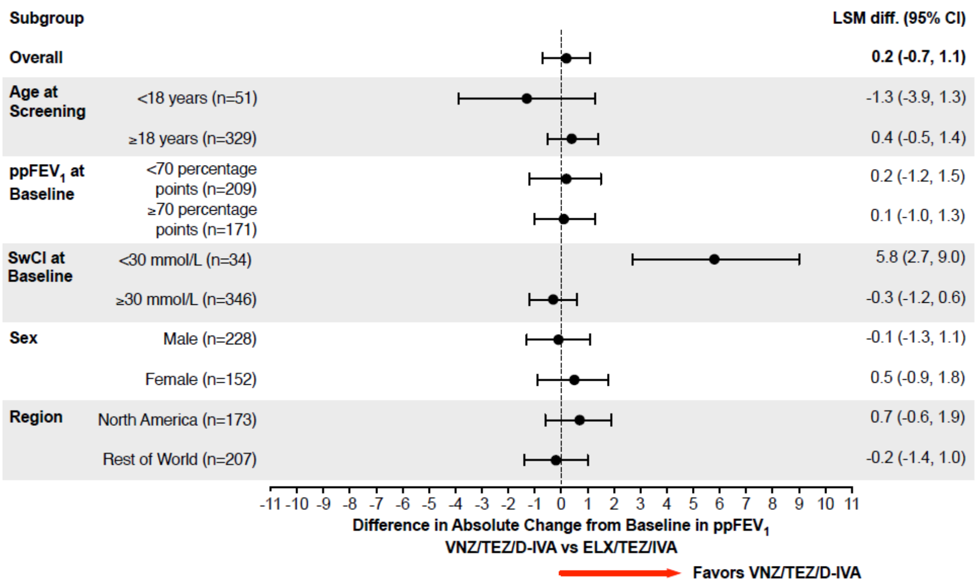

Trial VX20-121-103

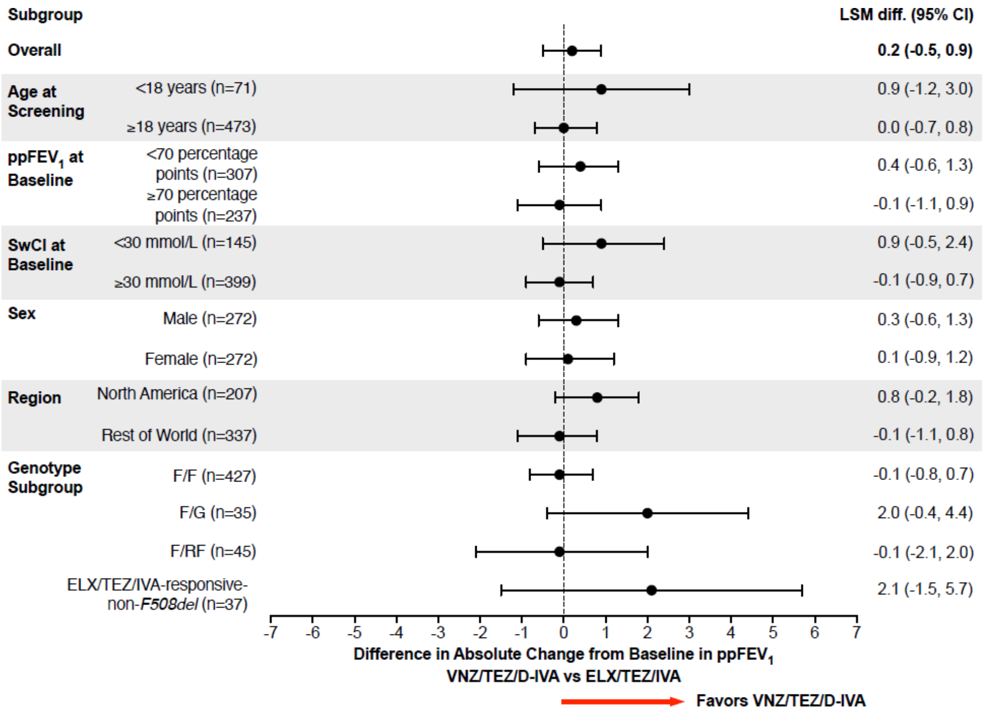

CI: confidence interval; ELX/TEZ/IVA: elexacaftor/tezacaftor/ivacaftor; F/F: homozygous for *F508del*; F/G: heterozygous for *F508del* and a gating mutation; F/MF: heterozygous for *F508del* and a minimal function mutation; F/RF: heterozygous for *F508del* and a residual function mutation; LSM: least squares mean; n: size of subsample; $ppFEV_1$: percent predicted forced expiratory volume in 1 second; SwCl: sweat chloride; VNZ/TEZ/D-IVA: vanzacaftor/tezacaftor/deutivacaftor

Figure S2. Time-to-First ALT/AST >3×ULN Event During the Treatment Emergent Period for the Treatment Period

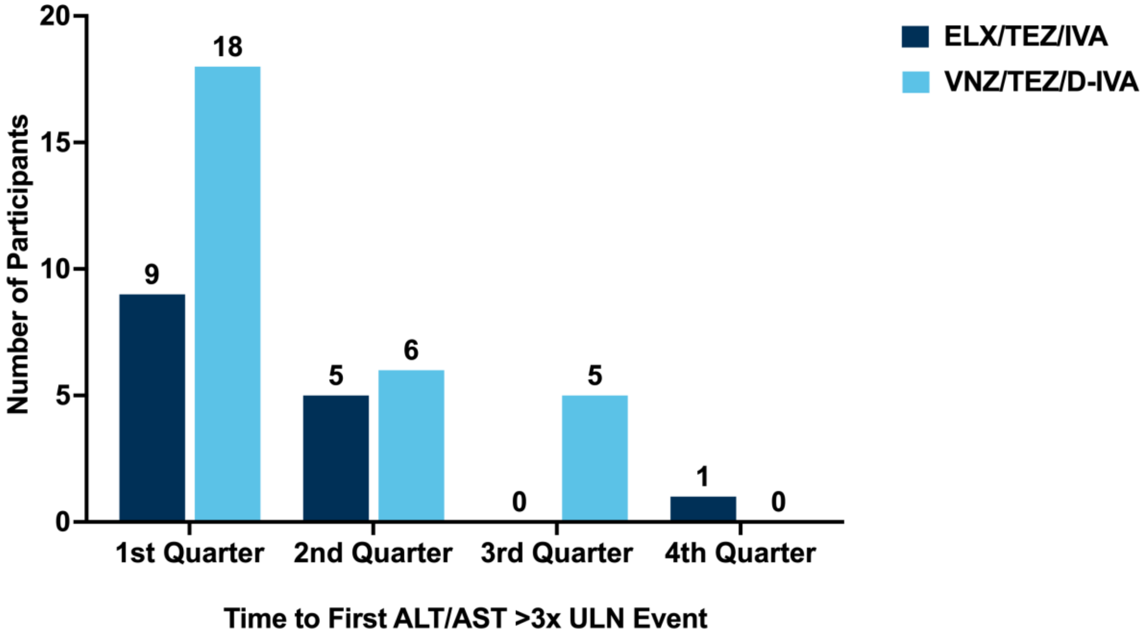

ALT: alanine transaminase; AST: aspartate transaminase; ELX/TEZ/IVA: elexacaftor/tezacaftor/ivacaftor; ULN: upper limit of normal; VNZ/TEZ/D-IVA: vanzacaftor/tezacaftor/deutivacaftor

Figure S3. Time-to-First Rash Event During the Treatment Emergent Period for Treatment Period

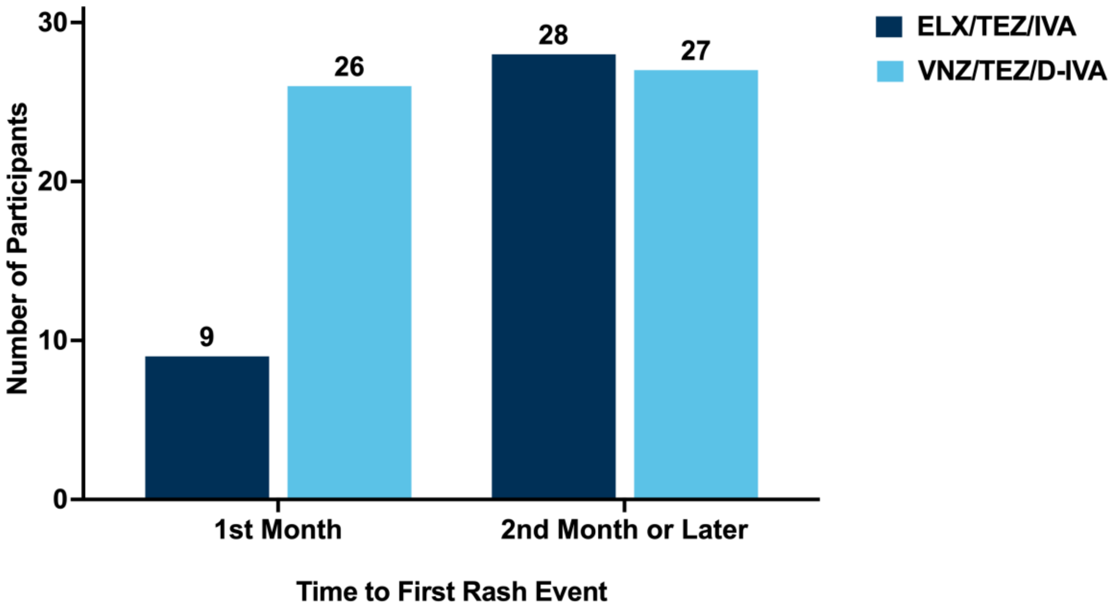

ELX/TEZ/IVA: elexacaftor/tezacaftor/ivacaftor; VNZ/TEZ/D-IVA: vanzacaftor/tezacaftor/deutivacaftor

Note: 1 participant in the ELX/TEZ/IVA group had a rash event without a complete start date. Therefore, they are counted in Table S17, but are not included in this figure.

Supplementary Tables

Table S1. Eligible Minimal Function *CFTR* Variants for Trial VX20-121-102.*

Q2X	E92X	Q290X	C524X	R709X	W846X	W1098X	S1255X
S4X	Q98X	G330X	Q525X	K710X	Y849X	R1102X	W1282X
W19X	Y122X	W401X	G542X	Q715X	R851X	E1104X	Q1313X
G27X	E193X	Q414X	G550X	L732X	Q890X	W1145X	Q1330X
Q39X	W216X	S434X	Q552X	R764X	S912X	R1158X	E1371X
W57X	L218X	S466X	R553X	R785X	Y913X	R1162X	Q1382X
E60X	Q220X	S489X	E585X	R792X	Q1042X	S1196X	Q1411X
R75X	Y275X	Q493X	G673X	E822X	W1089X	W1204X	
L88X	C276X	W496X	Q685X	W882X	Y1092X	L1254X	

185+1G→T	621+1G→T	1341+1G→A	1811+1.6kbA→G	2790-1G→C	3500-2A→G
296+1G→A	711+1G→T	1525-2A→G	1811+1643G→T	3040G→C (G970R)	3600+2insT
296+1G→T	711+5G→A	1525-1G→A	1812-1G→A	3120G→A	3850-1G→A
405+1G→A	712-1G→T	1717-8G→A	1898+1G→A	3120+1G→A	4005+1G→A
405+3A→C	1248+1G→A	1717-1G→A	1898+1G→C	3121-2A→G	4374+1G→T
406-1G→A	1249-1G→A	1811+1G→C	2622+1G→A	3121-1G→A	

182delT	574delA	1161delC	1609delCA	2184delA	2869insG	3271delGG	3905insT
306insA	663delT	1213delT	1677delTA	2184insA	2896insAG	3349insT	4016insT
365-366insT	849delG	1259insA	1782delA	2307insA	2942insT	3659delC	4021dupT
394delTT	935delA	1288insTA	1824delA	2347delG	2957delT	3737delA	4022insT
442delA	1078delT	1343delG	1833delT	2585delT	3007delG	3791delC	4040delA
444delA	1119delA	1471delA	2043delG	2594delGT	3028delA	3821delT	4279insA
457TAT→G	1138insG	1497delGG	2143delT	2711delT	3171delC	3876delA	4326delTC
541delC	1154insTC	1548delG	2183AA→G[†]	2732insA	3171insC	3878delG	

CFTRdele1	CFTRdele11	CFTRdele22-24	2055del9→A
CFTRdele2	CFTRdele13,14a	CFTRdele22,23	2105-2117del13insAGAAA
CFTRdele2,3	CFTRdele14b-17b	124del23bp	2372del8
CFTRdele2-4	CFTRdele16-17b	306delTAGA	2721del11
CFTRdele3-10,14b-16	CFTRdele17a,17b	602del14	2991del32
CFTRdele4-7	CFTRdele17a-18	852del22	3121-977_3499+248del2515
CFTRdele4-11	CFTRdele19	991del5	3667ins4
CFTR50kbdel	CFTRdele19-21	1461ins4	4010del4
CFTRdup6b-10	CFTRdele21	1924del7	4209TGTT→AA

A46D	I507del	R560S	R1066C
G85E	V520F	A561E	L1077P
R347P	A559T	Y569D	M1101K
L467P	R560T	L1065P	N1303K

* This list of qualifying variants was developed based on CFTR2.org.

[†] Also known as *2183delAA→G*.

Table S2. Trial VX20-121-102 Participant Enrollment by Country and Site

Country	Site name	Number of Participants Enrolled
United States	University of Alabama at Birmingham	1
	Nationwide Children's Hospital	3
	University of Washington Medical Center	3
	UPMC Children's Hospital of Pittsburgh	4
	University of Minnesota	4
	Children's Hospital of Philadelphia	1
	Washington University School of Medicine / St. Louis Children's Hospital	2
	The Emory Clinic at Chantilly	5
	Oregon Health & Science University	6
	CF Therapeutics Development Center of Western New York	2
	University of Utah	3
	The Children's Mercy Hospital	2
	Long Island Jewish Medical Center	7
	Nebraska Medical Center	3
	University of Virginia Health System	3
	Riley Hospital for Children at Indiana University Health	2
	University of Kentucky	2
	Massachusetts General Hospital Cystic Fibrosis Center Clinical Research Center	2
	Northwestern Memorial Hospital	3
	Helen DeVos Children's Hospital CF Center	4
	Vanderbilt University Medical Center	3
	Michigan Medicine	4
	National Jewish Health	6
	Texas Children's Hospital	2
	Indiana University	4
	Kaiser Permanente	1
	St. Luke's Cystic Fibrosis Center of Idaho	4
	Boston Children's Hospital	7
	ProMedica Toledo Hospital/Toledo Children's Hospital/Pediatric Pulmonary & Cystic Fibrosis Center	2
	Albany Medical College	2
	Cook Children's Health Care System	2
	Columbia University Medical Center	8
	Morristown Medical Center	4
	Dayton Children's Hospital	4
	Nemours Children's Hospital	1
	University of Wisconsin Hospital and Clinics	7
	Dartmouth Hitchcock Medical Center	4
	Children's Foundation Research Center / Le Bonheur Children's Hospital	1
	Vermont Lung Center	3
	UMass Memorial Medical Center	1
	New York Medical College	2
	Children's Hospital of Colorado	4
	The University of Texas Southwestern Medical Center	7
	Central Florida Pulmonary Group, PA	3
	Miller Children’s Hospital / Long Beach Memorial	5

Country	Site name	Number of Participants Enrolled
	Banner University of Arizona Medical Center	5
	Johns Hopkins All Children's Hospital Outpatient Care Center	2
	Children's Hospital Los Angeles	1
	University of Florida, Shands Hospital	2
	Joe DiMaggio Cystic Fibrosis & Pulmonary Center	5
	University of Kansas Medical Center	6
	Sanford Children's Speciality Clinic	3
	Cardinal Glennon Children's Hospital - St. Louis University	3
	University of Tennessee Medical Center	1
	Childrens Hospital University of Missouri Health Sciences Center	4
	Tulane Medical Center	3
	Augusta University	2
	Wake Forest Baptist Health	1
	Billings Clinic	1
	University of California San Francisco, Lung Transplant Program	3
	Atrium Health Levine Children's Hospital	2
	Driscoll Children's Hospital	1
	Inova Fairfax	1
United Kingdom	King's College Hospital	1
	St. Bartholomews Hospital	3
	Royal Papworth Hospital NHS Foundation Trust	3
	Royal Brompton Hospital	3
	All Wales Adult Cystic Fibrosis Centre, University Hospital Llandough	3
	Wythenshawe Hospital	6
	Clinical Research Facility	2
	University Hospitals Bristol and Weston NHS Foundation Trust, Bristol Royal Hospital	3
	Clinical Research Facility, Queen Elizabeth University Hospital	3
	Birmingham Heartlands Hospital	3
	Royal Devon University Healthcare NHS Foundation Trust	3
	St. James University Hospital	3
	Liverpool Heart and Chest Hospital	3
	Western General Hospital	3
Germany	Medizinische Hochschule Hannover	3
	Medizinische Hochschule Hannover	6
	Universitatsklinikum Wurzburg	3
	Mukoviszidose-Zentrum am Universitatsklinikum Jena, Klinik fur Kinder- und Jugendmedizin	3
	Friedrich-Alexander University of Erlangen-Nuremberg, University Children's Hospital	1
	St. Josef-Hospital	1
	Charite Paediatric Pulmonology Department	2
	Johann Wolfgang Goethe University	4
	Kinderklinik III, Abt. fur Pneumologie	2
	Ruhrlandklinik Westdeutsches Lungenzentrum am Klinikum Essen	19
	Pneumologisches Studienzentrum Muenchen-West	9
	Johannes Gutenberg-Universitaet	3
	Klinikum Westbrandenburg (CF)	4
	Universitatsklinikum Ulm, Klinik fur Kinder- und Jugendmedizin (CF)	3

Country	Site name	Number of Participants Enrolled
Sweden	Karolinska Universitetssjukhuset, Huddinge	7
	Sahlgrenska Universitetssjukhuset	6
	Lund University Skanes Universitetssjukhus	5
Ireland	University Hospital Limerick (Adults)	1
	Children's Health Ireland at Crumlin	1
	St. Vincent's University Hospital	2
	Cork University Hospital	1
	Children's Health Ireland at Temple Street	1
	Children's Health Ireland at Tallaght	1
Czech Republic	Fakultni nemocnice v Motole	5
	Klinika Detskych Infekcnich Nemoci	1
Hungary	National Koranyi Institute for TBC and Pulmonology	2
	Pulmonology Institute Torokbalint	11
Portugal	Hospital de Santa Maria	3
	Hospital Sao Joao	2
	CHP - Hospital de Santo Antonio	3
Spain	Hospital Universitario Virgen del Rocio	2
	Hospital Virgen de la Arrixaca	4
	Hospital Infantil Universitario Nino Jesus	2
	Corporacio Sanitaria Parc Tauli - Sabadell Hospital Universitari	3
	Hospital Universitario 12 de Octubre	2
	Hospital Universitari Vall d Hebron	2
	Hospital Universitario Ramon y Cajal	5
	Hospital Universitario y Politecnico La Fe	4
	Hospital Saint Joan de Deu	2
Israel	Sheba Medical Center - The Edmond and Lili Safra Children's Hospital	4
	Hadassah University Hospital Mount Scopus	6
	Schneider Children's Medical Center of Israel	7
New Zealand	Waikato Hospital	4
	Starship Children's Hospital	4
	Canterbury District Health Board	5
	Greenlane Clinical Centre	4
Australia	Royal Prince Alfred Hospital	4
	Mater Adult Hospital	1
	The Prince Charles Hospital	3
	Westmead Hospital	3
	Alfred Hospital	8

Table S3. Trial VX20-121-103 Participant Enrollment by Country and Site

Country	Site name	Number of Participants Enrolled
United States	University of Alabama at Birmingham	3
	Nationwide Children's Hospital	3
	University of Washington Medical Center	1
	UPMC Children's Hospital of Pittsburgh	3
	Children's Hospital of Philadelphia	3
	Washington University School of Medicine	4
	The Emory Clinic at Chantilly	2
	Oregon Health & Science University	4
	SUNY Upstate Medical University	1
	CF Therapeutics Development Center of Western New York	2
	University of Utah	3
	The Children's Mercy Hospital	2
	Long Island Jewish Medical Center	5
	University of Virginia Health System	3
	Riley Hospital for Children at Indiana University Health	3
	University of Kentucky	4
	Massachusetts General Hospital Cystic Fibrosis Center Clinical Research Center	9
	Northwestern Memorial Hospital	4
	Spectrum Health Medical Group Adult Cystic Fibrosis Care Center	3
	Vanderbilt University Medical Center	3
	West Virginia University	3
	Michigan Medicine	5
	National Jewish Health	3
	Indiana University	6
	St. Luke's Cystic Fibrosis Center of Idaho	7
	Boston Children's Hospital	6
	ProMedica Toledo Hospital/Toledo Children's Hospital/Pediatric Pulmonary & Cystic Fibrosis Center	4
	Maine Medical Partners	4
	Cook Children's Health Care System	6
	Columbia University Medical Center	6
	Morristown Medical Center	4
	Dayton Children's Hospital	2
	Akron Children's Hospital	3
	University Hospital and UW Health Clinics	4
	University of Oklahoma Health Sciences Center	2
	University of Miami Miller School of Medicine	2
	Vermont Lung Center	5
	UMass Memorial Medical Center	4
	Children's Hospital of Colorado	2
	The University of Texas Southwestern Medical Center	7
	Providence Pediatric Pulmonary & Cystic Fibrosis Clinic	4
	Harper University Hospital	4
	Central Florida Pulmonary Group, PA	3
	Banner University of Arizona Medical Center	8
	Yale New Haven Hospital	1

Country	Site name	Number of Participants Enrolled
	Johns Hopkins All Children's Hospital Outpatient Care Center	3
	Children's Hospital Los Angeles	3
	University of California Davis Medical Center	3
	Santiago Reyes, M.D.	1
	Nemours Children's Specialty Care	1
	Sanford Children's Speciality Clinic	2
	University of Tennessee Medical Center	1
	Tulane Medical Center	3
	University of Arkansas for Medical Sciences	6
	Wake Forest Baptist Health	1
	Billings Clinic	2
	Prisma Health Richland Campus	2
	Nemours Children's Specialty Care, Pensacola	2
	Children's Hospital of Orange County	2
Canada	Institut Universitaire de Cardiologie et Pneumologie de Quebec - Universite Laval	5
	Stollery Children's Hospital	2
	British Columbia Children's Hospital	3
	St. Paul's Hospital	3
	McGill University Health Centre, Glen Site, Montreal Children's Hospital	3
	University of Calgary Medical Clinic of the Foothills Medical Centre	5
	Centre Hospitalier de l'Universite de Montreal (CHUM) Hotel-Dieu	6
	St. Joseph's Health Care London	2
United Kingdom	Birmingham Children's Hospital	1
	King's College Hospital	1
	St. Bartholomew's Hospital	3
	Royal Manchester Children's Hospital	4
	Royal Papworth Hospital NHS Foundation Trust	3
	Royal Brompton Hospital	2
	All Wales Adult Cystic Fibrosis Centre, University Hospital Llandough	2
	Southampton General Hospital	3
	Wythenshawe Hospital	5
	Clinical Research Facility, Queen Elizabeth University Hospital	4
	St. James University Hospital	3
	Leeds General Infirmary	3
France	Centre Hospitalier Intercommunal Creteil	3
	Centre Hospitalier Universitaire (CHU) de Nice - Hopital Pasteur	6
	CHU de Rouen - Hopital Charles Nicolle	3
	Hopital Foch (Suresnes), Hopital Foch, Adultes	2
	Centre hospitalier universitaire de Nancy, Hopital de Braboi	1
	Hopital Robert Debre	3
	Centre de Perharidy	6
	Hopital Cochin	5
	Hopital Necker, Enfants Malades	1
	Hopital Arnaud de Villeneuve	3
	CHU Lyon - Hopital Femme Mere-Enfant	3
	Centre Hospitalier Lyon Sud	3
	Groupe Hospitaler Pellegrin, CHU De Bordeaux	3

Country	Site name	Number of Participants Enrolled
	Hôpital de Hautepierre, AX5	2
	Institut Cœur Poumon, CHU de Lille	3
	Hopital Bretonneau	3
Denmark	Aarhus University Hospital	3
	Juliane Marie Center, Rigshospitalet	8
Norway	Oslo University Hospital, Department of Paediatric Medicine	2
	Haukeland Universitetssjukehus (CF)	3
Sweden	Karolinska Universitetssjukhuset, Huddinge	7
	Sahlgrenska Universitetssjukhuset	7
	Lund University Skanes Universitetssjukhus	6
Austria	Medizinische Universität Wien	1
	University of Graz	2
	Medizinische Universität Innsbruck	4
	Uniklinikum Salzburg - Universitätsklinik für Pneumologie/Lungenheilkunde	3
Switzerland	Lindenhofspital - Quartier Bleu	3
	Kinderspital Zuerich	5
Italy	Malattie Apparato Respiratorio 2 - Centro Fibrosi Cistica	2
	Centro Regionale Fibrosi Cistica, A.O. Ospedale San Carlo	2
	Fondazione IRCCS Ospedale Maggiore Policlinico, Mangiagalli e Regina Elena	2
	Azienda Ospedaliero Universitaria Ospedale Pediatrico Meyer	3
	Azienda Ospedaliera di Verona - Ospedale Civile Maggiore	4
	Fondazione IRCCS Ca' Granda Ospedale Maggiore Policlinico	2
	Azienda Ospedaliero Universitaria Ospedale Riuniti	6
	IRCCS Istituto Giannina Gaslini-Ospedale Pediatrico	4
Belgium	Cliniques Universitaires Saint-Luc	4
	Universitaire Ziekenhuizen Leuven - Campus Gasthuisberg	6
	Universitair Ziekenhuis Gent	8
	Cliniques Universitaires de Bruxelles Hopital Erasme	5
	Universitair Ziekenhuis Brussel - Campus Jette	8
Poland	Klinika Mukowiscydozy IMD Oddzial Chorob Pluc Szpzoz IM. Dzieci WarszaWY	4
	Pediatric Hospital Polanki named of Maciej Płażyński	2
	Institute of Tuberculosis and Lung Diseases	7
Ireland	University Hospital Limerick (Adults)	1
	University Hospital Limerick (Pediatrics)	2
	Children's Health Ireland at Crumlin	1
	St. Vincent's University Hospital	2
	Cork University Hospital	4
	Children's University Hospital, Dublin	2
Germany	Friedrich-Alexander University of Erlangen-Nuremberg, University Children's Hospital	3
	Charite Paediatric Pulmonology Department	4
	Kinderklinik III, Abt. fur Pneumologie	1
	Ruhrlandklinik Westdeutsches Lungenzentrum am Klinikum Essen	25
	Justus-Liebig-Universität Gießen Zentrum für Kinderheilkunde und Jugendmedizin	3
	Pneumologisches Studienzentrum Muenchen-West	16
Netherlands	Erasmus Medical Center	3
	University Medical Center, Utrecht, Department of Pulmonology and Tuberculosis	1
	HagaZiekenhuis van den Haag	3

Country	Site name	Number of Participants Enrolled
	UMC St. Radboud	2
	Academisch Medisch Centrum (Academic Medical Centre)	4
Hungary	National Koranyi Institute for TBC and Pulmonology	6
	Pulmonology Institute Torokbalint	12
Greece	General Hospital of Thessaloniki 'Hippokratio', 3rd Pediatrics Department of Aristotle University of Thessaloniki	3
	General Hospital of Thessaloniki “Papanikolaou”	3
Israel	Sheba Medical Center - The Edmond and Lili Safra Children's Hospital	2
	Hadassah Medical Organization	2
	Schneider Children's Medical Center of Israel	1
Australia	Royal Prince Alfred Hospital	3
	The Royal Children's Hospital	1
	Mater Adult Hospital	2
	Queensland Children's Hospital	1
	The Prince Charles Hospital	4
	Institute for Respiratory Health	3
	Telethon Kids Institute	4
	Westmead Hospital	3
	Alfred Hospital	7
New Zealand	Waikato Hospital	4
	Starship Children's Hospital	1
	Canterbury District Health Board	6
	Greenlane Clinical Centre	14

Table S4. Trial VX20-121-102 List of IECs/ and IRBs and Approvals

Country	Institutional Review Board/Independent Ethics Committee	Approval Number
United States	Advarra, Inc.	PRO00054586
	The Children's Hospital of Philadelphia Institutional Review Board	IRB-21-018958
	University of Utah Institutional Review Board	IRB_00144008
	Partners Human Research Committee	2021P002821
	Northwestern University Office for Protection of Research Subjects	STU00215533
	Spectrum Health Research and Human Rights Committee	2021-226
	Vanderbilt Human Research Protection Program/ Institutional Review Board	211685
	Kaiser Permanente Northern California Institutional Review Board	00001045
	Committee on Clinical Investigation	IRB-P00038870
	Columbia University Medical Center IRB	IRB-AAAT8506
	Nemours IRB	1772359
	University of Vermont Research Protection Office	STUDY00001791
	John Hopkins Medicine All Children's Hospital Institutional Review Board	00290313
	Western Institutional Review Board	1326013
United Kingdom	North West - Heydock Research<br>Health Research Authority	IRAS 1003950<br>REC reference : 21/NW/0294
Germany	Ethikkommission der Universität Duisburg-Essen	45147 Essen
Ireland	National Office for Research and Ethics Committees (NREC)	21-NREC-CT-101
Czech Republic	Eticka Komise Pro Multicentricke Klinicke Hodnoceni Fakultni Nemocnice V Motole	EK-1130/21
	Eticka Komise FN Brno	80/21
Sweden	Etikprövningsmyndigheten	2021-05089
Hungary	Egeszsegugyi Tudomanyos Tanacs Klinikai Farmakologiai Etikai Bizottsaga	NIPN # OGYEI/13943-7/2022
Portugal	CEIC (Comissão de Ética para a Investigação Clínica)	20210851
Spain	CEIm Parc Tauli	NA
Israel	Helsinki Committee of the Chaim Sheba Medical Center	8634-21-SMC
	Hadassah University Hospital Ein Kerem	0545-21-HMO
	Helsinki Committee of Rabin Medical Center	0519-21-RMC
New Zealand	Southern Health and Disability Ethics Committee	2022 FULL 11901
Australia	Children's Health Queensland Hospital and Health Service Human Research Ethics Committee	HREC/21/QCHQ/78908

Table S5. Trial VX20-121-103 List of IECs/ and IRBs and Approvals

Country	Institutional Review Board/Independent Ethics Committee	Approval Number
United States	Advarra, Inc.	Pro00054721
	The Children's Hospital of Philadelphia Institutional Review Board	IRB 21-019108
	University of Utah Institutional Review Board	IRB_00144779
	UC Davis Institutional Review Board	2021P003087
	Northwestern University Office for Protection of Research Subjects	STU00215916
	Spectrum Health Research and Human Rights Committee	2021-227
	Vanderbilt Human Research Protection Program/ Institutional Review Board	#212033
	Committee on Clinical Investigation	#212033
	Columbia University Medical Center IRB	IRB-AAAT8507
	University of Vermont Research Protection Office	STUDY00001645
	John Hopkins Medicine All Children's Hospital Institutional Review Board	00290314
	Nemours IRB	1810970-3
Canada	Centre for Applied Ethics, McGill University Health Care	MP-37-2022-8254
	Human Research Ethics Board (HREB)	MS6_Pro00114251
	St. Michael's Hospital Research Ethics Board	21-291
	UBC Children's and Women's Research Ethics Board	H21-02371
	University of British Columbia Providence Health Care Research Ethics Board	H21- 03003
	The Conjoint Health Research Ethics Board (CHREB)	REB21-1645_MOD6
	Western University Health Science REB	2023-120066-77490
United Kingdom	North West- Greater Manchester South Research Ethics Committee	IRAS:1003951<br>REC Reference :21/NW/0307
Ireland	National Office for Research and Ethics Committees (NREC)	21-NREC-CT-100
Italy	Comitato Etico Territoriale Area Sud-Ovest Veneto	3518CESC
Austria	Ethikkommission der Medizinischen Universitaet Innsbruck	EK Nr:1364/2021
France	CPP Sud-Ouest et Outre-Mer IV	IS #: 21.03014.000028
Switzerland	Kantonale Ethikkommission Zürich	2021-02024
Netherlands	Medisch Ethische Toetsingscommissie (METC) UMC Utrecht	MvdL/mk/21/501143
Belgium	Universitair Ziekenhuis Leuven Campus Gasthuisberg Ethische Commissie Onderzoek	S65788
	Comité d'Ethique Cliniques Universitaires Saint-Luc	2021/21OCT/429
	Universitair Zeikenhuis Gent - Commissie voor Medische Ethiek	BC-11071
	Comite de ethique hospitalo-facultaire Erasme-ULB	SRB2021300 / P2021/531
	Universitair Ziekenhuis Brussel	2021/355
Denmark	De Videnskabsetiske Komiteer for Region Midtjylland	Case # 1-10-72-325-21
Poland	Komisja Bioetyczna Przy Instytcuie Matki i Dziecka, Ul	56/2021
Norway	REK sør-øst	310636
Sweden	Etikprövningsmyndigheten	2021-05156
Hungary	Egeszsegugyi Tudomanyos Tanacs Klinikai Farmakologiai Etikai Bizottsaga	OGYE-13961-2-2022
Greece	National Ethics Committee, Greek National Ethics Committee for Clinical Studies	NEC Code: 159 /21
Germany	Ethikkommission der Universität Duisburg-Essen	21-10319-AF
Israel	Helsinki Committee of the Chaim Sheba Medical Center	8635-21-SMC
	Hadassah University Hospital Ein Kerem	0546-21-HMO
	Helsinki Committee of Rabin Medical Center	0520-21-RMC
Australia	Children's Health Queensland Hospital and Health Service Human Research Ethics Committee	HREC/21/QCHQ/78521
	Bellberry Human Reserach Ethics Committee	2021-09-1054-A-3
New Zealand	Southern Health and Disability Ethics Committee	2022 FULL 12083

Table S6. Additional Baseline Characteristics.*

	Trial VX20-121-102		Trial VX20-121-103	
	**ELX/TEZ/IVA (N=202)**	**VNZ/TEZ/D-IVA (N=196)**	**ELX/TEZ/IVA (N=289)**	**VNZ/TEZ/D-IVA (N=284)**
***Pseudomonas aeruginosa* infection within 2 years prior to screening — n (%)**				
Positive	112 (55·4)	113 (57·7)	153 (52·9)	156 (54·9)
Negative	90 (44·6)	83 (42·3)	136 (47·1)	128 (45·1)
**Prior use of CFTR modulator therapy — n (%)†**				
Ivacaftor	0	1 (0·6)	5 (2·0)	11 (4·6)
Lumacaftor-ivacaftor	0	0	17 (6·8)	19 (7·9)
Tezacaftor-ivacaftor	0	1 (0·6)	24 (9·6)	26 (10·8)
Elexacaftor-tezacaftor-ivacaftor	177 (100·0)	168 (98·8)	204 (81·6)	185 (76·8)
**Prior use of dornase alfa — n (%)‡**				
Yes	136 (67·3)	123 (62·8)	179 (61·9)	177 (62·3)
No	66 (32·7)	73 (37·2)	110 (38·1)	107 (37·7)
**Prior use of azithromycin — n (%)‡**				
Yes	82 (40·6)	88 (44·9)	109 (37·7)	113 (39·8)
No	120 (59·4)	108 (55·1)	180 (62·3)	171 (60·2)
**Prior use of inhaled antibiotic — n (%)‡**				
Yes	91 (45·0)	81 (41·3)	106 (36·7)	93 (32·7)
No	111 (55·0)	115 (58·7)	183 (63·3)	191 (67·3)
**Prior use of any bronchodilator — n (%)‡**				
Yes	178 (88·1)	172 (87·8)	242 (83·7)	254 (89·4)
No	24 (11·9)	24 (12·2)	47 (16·3)	30 (10·6)
**Prior use of any inhaled hypertonic saline — n (%)‡**				
Yes	135 (66·8)	118 (60·2)	159 (55·0)	144 (50·7)
No	67 (33·2)	78 (39·8)	130 (45·0)	140 (49·3)
**BMI-for-age z-score — mean (SD) (participants aged ≤20 years)**	-0·14 (0·88)	-0·36 (1·09)	-0·30 (0·98)	-0·17 (0·95)

BMI: body mass index; CFTR: cystic fibrosis transmembrane conductance regulator; ELX/TEZ/IVA: elexacaftor/tezacaftor/ivacaftor; n: size of subsample; N: total sample size; SD: standard deviation; VNZ/TEZ/D-IVA: vanzacaftor/tezacaftor/deutivacaftor

* Baseline characteristics of the full analysis set (FAS), which was defined as all randomized participants who carried the intended *CFTR* mutations and received ≥1 dose of study drug in the treatment period. Baseline was defined as the most recent non-missing measurement before the first dose of study drug in the treatment period.

† Prior use was defined as any time within 56 days before the date of first dose in the treatment period, defined as the first dose of ELX/TEZ/IVA, IVA, or TEZ/IVA after randomization. This does not include ivacaftor or tezacaftor administered during the run-in period.

‡ Includes medications started 56 days prior to the first dose of study drug in the treatment period.

Table S7. Post-hoc Duration of ELX/TEZ/IVA Use as a Prior Medication (Pooled From Trials VX20-121-102 and VX20-121-103).

	ELX/TEZ/IVA N=491	VNZ/TEZ/D-IVA N=480	Total N=971
n	381	353	734
Mean (SD) (years)	1·96 (1·10)	2·06 (1·00)	2·01 (1·05)
Median	2·04	2·23	2·14
Interquartile range	1·52	1·30	1·44
Min, Max	0·13, 5·05	0·05, 5·07	0·05, 5·07

ELX/TEZ/IVA: elexacaftor/tezacaftor/ivacaftor; max: maximum; min: minimum; n: size of subsample; N: total sample size; SD: standard deviation; VNZ/TEZ/D-IVA: vanzacaftor/tezacaftor/deutivacaftor

Table S8. *CFTR* Variants Demonstrated to be Responsive Only to VNZ/TEZ/D-IVA in the FRT Assay.

3195del6	*A561E*	*G149R*	*I1234Vdel6aa*	*L1065P*	*Q1100P*	*R1066L*	*R560S*	*V520F*
3199del6	*A613T*	*G91R*	*I1398S*	*M1101R*	*Q452P*	*R1066M*	*R560T*	*Y569C*
A559T	*A72D*	*H199R*	*I506T*	*P99L*	*R1066C*	*R516G*	*T604I*	*Y913C*
A559V	*D513G*	*H609R*	*L102R*					

CFTR: cystic fibrosis transmembrane conductance regulator; FRT: Fischer Rat Thyroid; VNZ/TEZ/D-IVA: vanzacaftor/tezacaftor/deutivacaftor

Table S9. Post hoc Absolute Change in Sweat Chloride Concentration From Baseline Through Week 24 By Genotype in Trial VX20-121-103.

Genotype subgroup	ELX/TEZ/IVA N=289	VNZ/TEZ/D-IVA N=284
**Overall, n=546**		
LS mean change (SE)	-2·3 (0·7)	-5·1 (0·7)
LS mean difference, 95% CI	··	-2·8 (-4·7, -0·9)
***F508del/F508del*, n=427**		
LS mean change (SE)	-2·5 (0·8)	-5·7 (0·8)
LS mean difference, 95% CI	··	-3·2 (-5·3, -1·1)
***F508del*/gating, n=36**		
LS mean change (SE)	-2·4 (2·6)	3·6 (2·8)
LS mean difference, 95% CI	··	5·9 (-1·9, 13·7)
***F508del*/residual function, n=46**		
LS mean change (SE)	-1·0 (1·8)	-4·4 (1·8)
LS mean difference, 95% CI	··	-3·4 (-8·5, 1·6)
**ELX/TEZ/IVA-responsive-non-*F508del*, n=37**		
LS mean change (SE)	-2·1 (4·1)	-3·7 (4·5)
LS mean difference, 95% CI	··	-1·6 (-14·5, 11·2)

CI: confidence interval; ELX/TEZ/IVA: elexacaftor/tezacaftor/ivacaftor; LS: least squares; n: size of subsample; N: total sample size; SE: standard error; VNZ/TEZ/D-IVA: vanzacaftor/tezacaftor/deutivacaftor

Table S10. Post hoc Shift Tables for Proportion of Participants With Sweat Chloride <60 mmol/L Through Week 24 (Pooled from Trials VX20-121-102 and VX20-121-103)

	Baseline Category	N1	Through Week 24 Category: Missing n (%)	<60 mmol/L n (%)	≥60 mmol/L n (%)	<30 mmol/L n (%)	≥30 mmol/L n (%)
**ELX/TEZ/IVA**	<60 mmol/L	358	6 (1·7)	317 (88·5)	35 (9·8)	106 (29·6)	246 (68·7)
	≥60 mmol/L	125	3 (2·4)	46 (36·8)	76 (60·8)	1 (0·8)	121 (96·8)
	Total	483	9 (1·9)	363 (75·2)	111 (23·0)	107 (22·2)	367 (76·0)
**VNZ/TEZ/D-IVA**	<60 mmol/L	361	10 (2·8)	338 (93·6)	13 (3·6)	140 (38·8)	211 (58·4)
	≥60 mmol/L	115	4 (3·5)	58 (50·4)	53 (46·1)	1 (0·9)	110 (95·7)
	Total	476	14 (2·9)	396 (83·2)	66 (13·9)	141 (29·6)	321 (67·4)

ELX/TEZ/IVA: elexacaftor/tezacaftor/ivacaftor; N1: total sample size; n: size of subsample; VNZ/TEZ/D-IVA: vanzacaftor/tezacaftor/deutivacaftor

Note: Proportion of participants with sweat chloride <60 mmol/L through week 24 was evaluated for participants with non-missing baseline.

Table S11. Post hoc Subgroup Analysis of Absolute Change From Baseline in Sweat Chloride (mmol/L) Through Week 24 for Participants With Sweat Chloride <30 mmol/L Through Week 24

	Overall		Shift from any baseline to <30 mmol/L through week 24		Shift from ≥30 mmol/L at baseline to <30 mmol/L through week 24	
	**ELX/TEZ/IVA**	**VNZ/TEZ/D-IVA**	**ELX/TEZ/IVA**	**VNZ/TEZ/D-IVA**	**ELX/TEZ/IVA**	**VNZ/TEZ/D-IVA**
**Trial VX20-121-102**	**N = 202**	**N = 196**	**N = 19**	**N = 43**	**N = 9**	**N = 29**
Baseline sweat chloride; mean (SD)	54·3 (18·2)	53·6 (17·0)	32·3 (3·7)	36·8 (1·8)	44·2 (5·4)	42·7 (1·7)
Absolute change through week 24						
LS mean change (SE)	0·9 (0·8)	-7·5 (0·8)	-7·0 (1·4)	-10·7 (0·8)	-10·7 (2·1)	-15·6 (1·0)
LS mean difference, 95% CI	--	-8·4 (-10·5, -6·3)	--	-3·7 (-6·9, -0·5)	--	-4·9 (-9·8, 0·0)
**Trial VX20-121-103**	**N = 289**	**N = 284**	**N = 101**	**N = 114**	**N = 27**	**N = 49**
Baseline sweat chloride; mean (SD)	42·1 (17·9)	43·4 (18·5)	25·9 (1·1)	28·9 (1·2)	41·0 (2·1)	39·7 (1·6)
Absolute change through week 24						
LS mean change (SE)	-2·3 (0·7)	-5·1 (0·7)	-5·0 (0·5)	-6·9 (0·5)	-13·9 (1·0)	-16·5 (0·7)
LS mean difference, 95% CI	--	-2·8 (-4·7, -0·9)	--	-1·8 (-3·3, -0·4)	--	-2·7 (-5·2, -0·1)

ELX/TEZ/IVA: elexacaftor/tezacaftor/ivacaftor; LS: least squares; N: total sample size; SE: standard error; VNZ/TEZ/D-IVA: vanzacaftor/tezacaftor/deutivacaftor

Table S12. Post hoc Shift Tables for Proportion of Participants With Sweat Chloride <30 mmol/L Through Week 24 (Pooled From Trials VX20-121-102 and VX20-121-103)

			Through Week 24 Category		
	**Baseline Category**	**N1**	**Missing n (%)**	**<30 mmol/L n (%)**	**≥30 mmol/L n (%)**
**ELX/TEZ/IVA**	<30 mmol/L	99	2 (2·0)	82 (82·8)	15 (15·2)
	≥30 mmol/L	384	7 (1·8)	25 (6·5)	352 (91·7)
	Total	483	9 (1·9)	107 (22·2)	367 (76·0)
**VNZ/TEZ/D-IVA**	<30 mmol/L	89	3 (3·4)	76 (85·4)	10 (11·2)
	≥30 mmol/L	387	11 (2·8)	65 (16·8)	311 (80·4)
	Total	476	14 (2·9)	141 (29·6)	321 (67·4)

ELX/TEZ/IVA: elexacaftor/tezacaftor/ivacaftor; N1: total sample size; n: size of subsample; VNZ/TEZ/D-IVA: vanzacaftor/tezacaftor/deutivacaftor

Note: Proportion of participants with sweat chloride <30 mmol/L through week 24 was evaluated for participants with non-missing baseline.

Table S13. Other Secondary Efficacy Endpoints.

	Trial VX20-121-102		Trial VX20-121-103	
	ELX/TEZ/IVA (N=202)	VNZ/TEZ/D-IVA (N=196)	ELX/TEZ/IVA (N=289)	VNZ/TEZ/D-IVA (N=284)
**Absolute change in CFQ-R RD score from baseline through week 24**				
Mean (SD)	-1·7 (1·0)	0·5 (1·1)	-1·2 (0·8)	-1·2 (0·8)
LS mean difference, 95% CI	··	2·3 (-0·6, 5·2)	··	-0·1 (-2·3, 2·1)
**Absolute change in sweat chloride concentration from baseline through week 52**				
Mean (SD)	0·5 (0·7)	-7·5 (0·7)	-2·2 (0·6)	-5·0 (0·6)
LS mean difference, 95% CI	··	-8·0 (-9·9, -6·1)	··	-2·8 (-4·6, -1·0)
**Absolute change in $ppFEV_1$ from baseline through week 52**				
Mean (SD)	0·4 (0·3)	0·5 (0·3)	0·0 (0·2)	0·3 (0·2)
LS mean difference, 95% CI	··	0·1 (-0·8, 1·0)	··	0·3 (-0·4, 1·0)
**Proportion of participants with sweat chloride concentration <60 mmol/L from baseline through week 24 (individual trial)**				
Proportion	0·59	0·81	0·89	0·90
Odds ratio†, 95% CI	··	4·28 (2·57, 7·11)	··	1·10 (0·65, 1·87)
**Proportion of participants with sweat chloride concentration <30 mmol/L from baseline through week 24 (individual trial)**				
Proportion	0·07	0·20	0·34	0·38
Odds ratio†, 95% CI	··	7·19 (3·54, 14·6)	··	2·06 (1·33, 3·18)
**Number of pulmonary exacerbations through week 52**				
Annual event rate	0·42	0·32	0·26	0·29
Rate difference, 95% CI	··	-0·10 (-0·24, 0·04)	··	0·03 (-0·07, 0·13)

CFQ-R: Cystic Fibrosis Questionnaire–Revised; CI: confidence interval; ELX/TEZ/IVA: elexacaftor/tezacaftor/ivacaftor; LS: least squares; N: total sample size; $ppFEV_1$: percent predicted forced expiratory volume in 1 second; RD: respiratory domain; SD: standard deviation; VNZ/TEZ/D-IVA: vanzacaftor/tezacaftor/deutivacaftor

Note: Baseline was after the run-in period and was defined as the most recent non-missing measurement before the first dose of study drug in the treatment period.

† The GEE model was used to estimate the odds ratio. Observed proportion is presented.

Table S14. Serious Adverse Events That Occurred in Two or More Participants in Either Group in Pooled Trials VX20-121-102 and VX20-121-103.

Preferred Term	Trials VX20-121-102 and VX20-121-103 ELX/TEZ/IVA N=491 n (%)	VNZ/TEZ/D-IVA N=480 n (%)
Any serious adverse event	81 (16·5)	68 (14·2)
Infective pulmonary exacerbation of cystic fibrosis	35 (7·1)	29 (6·0)
Influenza	3 (0·6)	7 (1·5)
Hemoptysis	3 (0·6)	3 (0·6)
Pneumonia	6 (1·2)	4 (0·8)
Suicidal ideation	2 (0·4)	2 (0·4)
Syncope	0	2 (0·4)
COVID-19	4 (0·8)	2 (0·4)
DIOS	3 (0·6)	2 (0·4)
ALT increased	2 (0·4)	2 (0·4)
AST increased	1 (0·2)	2 (0·4)
Depression	0	2 (0·4)
GGT increased	1 (0·2)	2 (0·4)
Cholelithiasis	3 (0·6)	0
Constipation	3 (0·6)	0
Nephrolithiasis	2 (0·4)	0

ALT: alanine transaminase; AST: aspartate transaminase; COVID-19: coronavirus disease 2019; DIOS: distal intestinal obstruction syndrome; ELX/TEZ/IVA: elexacaftor/tezacaftor/ivacaftor; GGT: gamma-glutamyl transferase; n: size of subsample; N: total sample size; VNZ/TEZ/D-IVA: vanzacaftor/tezacaftor/deutivacaftor

Table S15. Adverse Events That Led to Treatment Discontinuation in at Least 2 Participants in Either Group in Pooled Trials VX20-121-102 and VX20-121-103.

Preferred Term	Trials VX20-121-102 and VX20-121-103 ELX/TEZ/IVA N=491 n (%)	VNZ/TEZ/D-IVA N=480 n (%)
Any adverse events leading to treatment discontinuation	18 (3·7)	18 (3·8)
ALT increased	3 (0·6)	7 (1·5)
AST increased	3 (0·6)	6 (1·3)
Blood bilirubin increased	0	2 (0·4)
Blood bilirubin unconjugated increased	0	2 (0·4)
Fatigue	2 (0·4)	3 (0·6)
Blood alkaline phosphatase increased	1 (0·2)	2 (0·4)
Cough	1 (0·2)	2 (0·4)
Depression	0	2 (0·4)
Anxiety	0	2 (0·4)
Dysphonia	0	2 (0·4)
Pulmonary function test decreased	2 (0·4)	0

ALT: alanine transaminase; AST: aspartate transaminase; ELX/TEZ/IVA: elexacaftor/tezacaftor/ivacaftor; n: size of subsample; N: total sample size; VNZ/TEZ/D-IVA: vanzacaftor/tezacaftor/deutivacaftor

Table S16. Summary of Elevated Transaminases Events and Liver Function Test Enzyme Elevations in Pooled Trials VX20-121-102 and VX20-121-103.†

	Trials VX20-121-102 and VX20-121-103		Trials VX17-445-102 and VX17-445-105 (Post-hoc analyses of 52-week data)
	**ELX/TEZ/IVA N=491 n (%)**	**VNZ/TEZ/D-IVA N=480 n (%)**	**ELX/TEZ/IVA N=403 n (%)**
**Participants with any elevated transaminase events**	35 (7·1)	43 (9·0)	52 (12·9)
**Maximum severity of adverse event**			
Mild	20 (4·1)	24 (5·0)	29 (7·2)
Moderate	13 (2·6)	15 (3·1)	18 (4·5)
Severe	2 (0·4)	4 (0·8)	5 (1·2)
Life-threatening	0	0	0
**Participants with adverse events leading to discontinuation of trial regimen**	3 (0·6)	7 (1·5)	4 (1·0)
**Participants with adverse events leading to interruption of trial regimen**	3 (0·6)	5 (1·0)	11 (2·7)
**Participants with serious adverse events**	2 (0·4)	2 (0·4)	3 (0·7)
ALT/AST*			
>3×ULN	15 (3·1)	29 (6·0)	38 (9·4)
>5×ULN	6 (1·2)	12 (2·5)	16 (4·0)
>8×ULN	1 (0·2)	6 (1·3)	6 (1·5)
**ALT/AST >3×ULN & total bilirubin >2×ULN****	2 (0·4)	1 (0·2)	2 (0·5)
**ALT/AST >3×ULN with concurrent, newly occurring total bilirubin >2×ULN**	0	1 (0·2)‡	0

ALT: alanine aminotransferase; AST: aspartate aminotransferase; ELX/TEZ/IVA: elexacaftor/tezacaftor/ivacaftor; n: size of subsample; N: total sample size; ULN: upper limit of normal; VNZ/TEZ/D-IVA: vanzacaftor/tezacaftor/deutivacaftor

* For the liver function test threshold analyses, each percentage is calculated as (n/N1)×100, where the numerator n is the number of participants meeting the indicated threshold and the denominator (N1) is the number of participants with at least one non-missing measurement during the treatment-emergent period. For "ALT or AST", counts are based on the highest value of either test during the treatment-emergent period for each participant. A participant whose highest value is >5×ULN is also counted as >3×ULN. A participant whose highest value is >8×ULN is also counted as >3×ULN and >5×ULN.

** These ALT/AST elevations were not concurrent with bilirubin elevations.

† Group term of "elevated transaminase events" included multiple Preferred Terms; those that occurred in this study were alanine aminotransferase increased, aspartate aminotransferase increased, and liver function test increased.

‡ Increase in indirect bilirubin only, consistent with Gilbert's syndrome (confirmed by genetic testing).

Table S17. Summary of Rash Events in Pooled Trials VX20-121-102 and VX20-121-103.*

	Trials VX20-121-102 and VX20-121-103		Trials VX17-445-102 and VX17-445-105 (Post-hoc analyses of 52-week data)
	**ELX/TEZ/IVA N=491 n (%)**	**VNZ/TEZ/D-IVA N=480 n (%)**	**ELX/TEZ/IVA N = 403 n (%)**
**Participants with any rash events**	38 (7·7)	53 (11·0)	65 (16·1)
**Maximum severity of adverse event**			
Mild	27 (5·5)	39 (8·1)	45 (11·2)
Moderate	11 (2·2)	13 (2·7)	18 (4·5)
Severe	0	1 (0·2)	2 (0·5)
**Participants with adverse events leading to discontinuation of trial regimen**	0	1 (0·2)	2 (0·5)
**Participants with adverse events leading to interruption of trial regimen**	0	2 (0·4)	9 (2·2)
**Participants with serious adverse events**	0	0	4 (1·0)

ELX/TEZ/IVA: elexacaftor/tezacaftor/ivacaftor; n: size of subsample; N: total sample size; VNZ/TEZ/D-IVA: vanzacaftor/tezacaftor/deutivacaftor

* When summarizing numbers and percentages of participants, a participant with multiple events within a category is counted only once in that category. Group term of "rash events" included multiple Preferred Terms including, rash, rash erythematous, rash maculopapular, rash papular, skin exfoliation, and urticaria.

Table S18. Summary of CK Elevation Events in Pooled Trials VX20-121-102 and VX20-121-103.

	Trials VX20-121-102 and VX20-121-103	
	**ELX/TEZ/IVA N = 491 n (%)**	**VNZ/TEZ/D-IVA N = 480 n (%)**
**Participants with any CK elevation events**	41 (8·4)	43 (9·0)
**Maximum severity of adverse events**		
Mild	28 (5·7)	30 (6·3)
Moderate	11 (2·2)	12 (2·5)
Severe	2 (0·4)	1 (0·2)
Life-threatening	0	0
**Participants with adverse events leading to discontinuation of trial regimen**	1 (0·2)	1 (0·2)
**Participants with adverse events leading to interruption of trial regimen**	1 (0·2)	1 (0·2)
**Participants with serious adverse events**	1 (0·2)	1 (0·2)

CK: creatine kinase; ELX/TEZ/IVA: elexacaftor/tezacaftor/ivacaftor; n: size of subsample; N: total sample size; VNZ/TEZ/D-IVA: vanzacaftor/tezacaftor/deutivacaftor

Table S19. Summary of Neuropsychiatric Events in Pooled Trials VX20-121-102 and VX20-121-103.*

	Trials VX20-121-102 and VX20-121-103	
	**ELX/TEZ/IVA N=491 n (%)**	**VNZ/TEZ/D-IVA N=480 n (%)**
**Participants with any neuropsychiatric adverse events**	59 (12·0)	55 (11·5)
**Maximum severity of adverse events**		
Mild	26 (5·3)	28 (5·8)
Moderate	27 (5·5)	23 (4·8)
Severe	6 (1·2)	4 (0·8)
Life-threatening	0	0
**Participants with adverse events leading to discontinuation of trial regimen**	2 (0·4)	3 (0·6)
**Participants with adverse events leading to interruption of trial regimen**	3 (0·6)	2 (0·4)
**Participants with serious adverse events**	4 (0·8)	4 (0·8)

ELX/TEZ/IVA: elexacaftor/tezacaftor/ivacaftor; n: size of subsample; N: total sample size; VNZ/TEZ/D-IVA: vanzacaftor/tezacaftor/deutivacaftor

* When summarizing numbers and percentages of participants, a participant with multiple events within a category is counted only once in that category. Group term of "neuropsychiatric events" included multiple Preferred Terms, including depression/suicidality, anxiety, insomnia, behavioral, mental fatigue, etc.

Table S20. Neuropsychiatric Adverse Events in Pooled Trials VX20-121-102 and VX20-121-103.

	Trials VX20-121-102 and VX20-121-103	
**Preferred Term**	**ELX/TEZ/IVA N=491 n (%)**	**VNZ/TEZ/D-IVA N=480 n (%)**
**Participants with any neuropsychiatric adverse event**	59 (12·0)	55 (11·5)
Adjustment disorder with anxiety	1 (0·2)	0
Adjustment disorder with depressed mood	1 (0·2)	0
Anger	2 (0·4)	1 (0·2)
Anhedonia	1 (0·2)	0
Anxiety	10 (2·0)	21 (4·4)
Attention deficit hyperactivity disorder	4 (0·8)	1 (0·2)
Behaviour disorder	1 (0·2)	0
Brain fog	0	1 (0·2)
Depressed mood	7 (1·4)	4 (0·8)
Depression	10 (2·0)	14 (2·9)
Depression suicidal	1 (0·2)	0
Depressive symptom	3 (0·6)	0
Disturbance in attention	3 (0·6)	0
Generalised anxiety disorder	1 (0·2)	0
Initial insomnia	1 (0·2)	3 (0·6)
Insomnia	22 (4·5)	15 (3·1)
Irritability	2 (0·4)	1 (0·2)
Major depression	0	1 (0·2)
Memory impairment	1 (0·2)	1 (0·2)
Mental fatigue	1 (0·2)	2 (0·4)
Middle insomnia	1 (0·2)	2 (0·4)
Mixed anxiety and depressive disorder	1 (0·2)	0
Physical assault	0	1 (0·2)
Suicidal ideation	4 (0·8)	2 (0·4)

ELX/TEZ/IVA: elexacaftor/tezacaftor/ivacaftor; n: size of subsample; N: total sample size; VNZ/TEZ/D-IVA: vanzacaftor/tezacaftor/deutivacaftor

Table S21. Post hoc Summary: Depression-related Events in Pooled Trials VX20-121-102 and VX20-121-103.*

	Trials VX20-121-102 and VX20-121-103	
	**ELX/TEZ/IVA N=491 n (%)**	**VNZ/TEZ/D-IVA N=480 n (%)**
**Participants with any depression-related adverse events**	25 (5·1)	20 (4·2)
**Maximum severity of adverse events**		
Mild	12 (2·4)	7 (1·5)
Moderate	10 (2·0)	9 (1·9)
Severe	3 (0·6)	4 (0·8)
Life-threatening	0	0
**Adverse events leading to discontinuation of trial regimen**	1 (0·2)	2 (0·4)
**Adverse events leading to interruption of trial regimen**	1 (0·2)	1 (0·2)
**Serious adverse events**	3 (0·6)	4 (0·8)

ELX/TEZ/IVA: elexacaftor/tezacaftor/ivacaftor; n: size of subsample; N: total sample size; VNZ/TEZ/D-IVA: vanzacaftor/tezacaftor/deutivacaftor

* When summarizing numbers and percentages of participants, a participant with multiple events within a category is counted only once in that category. Group term of "depression-related" included multiple Preferred Terms.

Table S22. Summary of Blood Pressure Measurements.

Mean (SD), mmHg	Trial VX20-121-102				Trial VX20-121-103			
	**ELX/TEZ/IVA N=202**		**VNZ/TEZ/D-IVA N=196**		**ELX/TEZ/IVA N=289**		**VNZ/TEZ/D-IVA N=284**	
	**SBP**	**DBP**	**SBP**	**DBP**	**SBP**	**DBP**	**SBP**	**DBP**
**Baseline***	117·2 (11·3)	72·5 (9·0)	116·6 (12·5)	72·5 (9·5)	117·8 (12·1)	72·7 (9·9)	118·5 (12·3)	73·5 (9·4)
Δ Day 15	0·4 (10·6)	0·2 (7·6)	-0·1 (10·1)	0·3 (8·8)	0·8 (10·9)	0·3 (8·4)	-0·4 (10·8)	-0·8 (8·6)
Δ Week 4	0·3 (10·1)	0·3 (7·2)	0·1 (11·8)	-0·4 (8·8)	0·9 (10·7)	0·1 (9·0)	-0·5 (10·3)	-1·2 (8·2)
Δ Week 8	0·7 (11·5)	1·0 (8·1)	0·2 (11·3)	-0·2 (9·0)	1·4 (10·0)	0·5 (9·0)	0·2 (10·8)	-0·6 (8·9)
Δ Week 12	1·3 (11·5)	1·0 (8·3)	1·8 (11·4)	0·4 (8·8)	2·0 (10·8)	1·3 (8·7)	1·3 (11·2)	-0·2 (8·7)
Δ Week 16	0·9 (9·9)	1·0 (7·6)	0·9 (10·0)	-0·3 (9·7)	1·4 (10·8)	0·4 (8·3)	0·4 (11·6)	-0·7 (9·0)
Δ Week 24	0·8 (10·4)	1·0 (8·1)	0 (10·9)	-0·4 (9·0)	1·8 (11·2)	0·8 (8·9)	0·1 (10·7)	-1·5 (8·7)
Δ Week 36	1·3 (10·8)	1·1 (7·6)	1·4 (10·7)	-0·1 (8·7)	1·8 (12·0)	0·9 (9·0)	0·8 (10·5)	-0·3 (8·5)
Δ Week 52	1·6 (10·6)	0·7 (8·2)	0·3 (11·3)	-0·5 (9·2)	2·6 (11·1)	1·6 (9·9)	0·4 (11·9)	-0·7 (10·2)

DBP: diastolic blood pressure; ELX/TEZ/IVA: elexacaftor/tezacaftor/ivacaftor; n: size of subsample; N: total sample size; SBP: systolic blood pressure; SD: standard deviation; VNZ/TEZ/D-IVA: vanzacaftor/tezacaftor/deutivacaftor

* Baseline was defined as the most recent non-missing measurement before the first dose of study drug in the treatment period.

References

1. Van Goor F, Yu H, Burton B, Hoffman BJ. Effect of ivacaftor on CFTR forms with missense mutations associated with defects in protein processing or function. J Cyst Fibros. 2014;13(1):29-36.

2. Yu H, Burton B, Huang CJ, et al. Ivacaftor potentiation of multiple CFTR channels with gating mutations. J Cyst Fibros. 2012;11(3):237-45.

3. Van Goor F, Straley KS, Cao D, et al. Rescue of deltaF508-CFTR trafficking and gating in human cystic fibrosis airway primary cultures by small molecules. Am J Physiol Lung Cell Mol Physiol. 2006;290(6):L1117-30.

4. Durmowicz AG, Lim R, Rogers H, Rosebraugh CJ, Chowdhury BA. The U.S. Food and Drug Administration's experience with ivacaftor in cystic fibrosis. Establishing efficacy using in vitro data in lieu of a clinical trial. Ann Am Thorac Soc. 2018;15(1):1-2.

5. Quanjer PH, Stanojevic S, Cole TJ, et al. Multi-ethnic reference values for spirometry for the 3-95-yr age range: the global lung function 2012 equations. Eur Respir J. 2012;40(6):1324-43.

6. Graham BL, Steenbruggen I, Miller MR, et al. Standardization of spirometry 2019 update. An official American Thoracic Society and European Respiratory Society technical statement. Am J Respir Crit Care Med. 2019;200(8):e70-e88.

7. Levey AS, Bosch JP, Lewis JB, Greene T, Rogers N, Roth D. A more accurate method to estimate glomerular filtration rate from serum creatinine: a new prediction equation. Modification of Diet in Renal Disease Study Group. Ann Intern Med. 1999;130(6):461-70.

8. Levey AS, Coresh J, Greene T, et al. Using standardized serum creatinine values in the modification of diet in renal disease study equation for estimating glomerular filtration rate. Ann Intern Med. 2006;145(4):247-54.

9. Counahan R, Chantler C, Ghazali S, Kirkwood B, Rose F, Barratt TM. Estimation of glomerular filtration rate from plasma creatinine concentration in children. Arch Dis Child. 1976;51(11):875-8.

10. Kenward MG, Roger JH. Small sample inference for fixed effects from restricted maximum likelihood. Biometrics. 1997;53(3):983-97.

11. Fuchs HJ, Borowitz DS, Christiansen DH, et al. Effect of aerosolized recombinant human DNase on exacerbations of respiratory symptoms and on pulmonary function in patients with cystic fibrosis. N Engl J Med. 1994;331(10):637-42.

12. Ramsey BW, Davies J, McElvaney NG, et al. A CFTR potentiator in patients with cystic fibrosis and the G551D mutation. New England Journal of Medicine. 2011;365(18):1663-72.
